# Adipose-targeted triiodothyronine therapy counteracts obesity-related metabolic complications and atherosclerosis with negligible side effects

**DOI:** 10.1038/s41467-022-35470-4

**Published:** 2022-12-20

**Authors:** Kang Chen, Lai Yee Cheong, Yuan Gao, Yaming Zhang, Tianshi Feng, Qin Wang, Leigang Jin, Eric Honoré, Karen S. L. Lam, Weiping Wang, Xiaoyan Hui, Aimin Xu

**Affiliations:** 1 State Key Laboratory of Pharmaceutical Biotechnology, The University of Hong Kong, 21 Sassoon Road, Laboratory Block, Pokfulam, Hong Kong China; 2 Department of Medicine, Li Ka Shing Faculty of Medicine, The University of Hong Kong, Hong Kong, China; 3 Dr Li Dak-Sum Research Centre, The University of Hong Kong-Karolinska Institutet Collaboration in Regenerative Medicine, The University of Hong Kong, Pokfulam, Hong Kong China; 4 Department of Pharmacology & Pharmacy, Li Ka Shing Faculty of Medicine, The University of Hong Kong, Pokfulam, Hong Kong China; 5 Université Côte d’Azur, Centre National de la Recherche Scientifique, Institut National de la Santé et de la Recherche Médicale, Institut de Pharmacologie Moléculaire et Cellulaire, Labex ICST, Valbonne, France

**Keywords:** Adipocytes, Drug therapy, Endocrine system and metabolic diseases

## Abstract

Thyroid hormone (TH) is a thermogenic activator with anti-obesity potential. However, systemic TH administration has no obvious clinical benefits on weight reduction. Herein we selectively delivered triiodothyronine (T3) to adipose tissues by encapsulating T3 in liposomes modified with an adipose homing peptide (PLT3). Systemic T3 administration failed to promote thermogenesis in brown and white adipose tissues (WAT) due to a feedback suppression of sympathetic innervation. PLT3 therapy effectively obviated this feedback suppression on adrenergic inputs, and potently induced browning and thermogenesis of WAT, leading to alleviation of obesity, glucose intolerance, insulin resistance, and fatty liver in obese mice. Furthermore, PLT3 was much more effective than systemic T3 therapy in reducing hypercholesterolemia and atherosclerosis in apoE-deficient mice. These findings uncover WAT as a viable target mediating the therapeutic benefits of TH and provide a safe and efficient therapeutic strategy for obesity and its complications by delivering TH to adipose tissue.

## Abstract (web summary)

Although thyroid hormone (TH) has anti-obesity potential, systemic administration of TH causes severe adverse effects without obvious weight loss. Here, the authors show that adipose tissue-targeted delivery of TH with liposomes is a safe and efficient strategy to treat obesity and its related complications in mice.

## Introduction

The prevalence of overweight and obesity, defined as excess accumulation of adipose tissues, has reached epidemic proportions globally^1,2^. Obesity is a significant risk factor for a myriad of chronic diseases, inducing type 2 diabetes, fatty liver, cardiovascular diseases, neurodegenerative disorders, osteoarthritis, sleep apnea, and certain types of cancer^3,4^. However, pharmacological options for treatment of obesity remain limited^5^. Most of the anti-obesity medications, especially those acting centrally to suppress food intake, were withdrawn from the market due to their severe adverse effects. Recently, there is considerable interest in targeting thermogenesis to boost energy expenditure for weight loss, but no efficient and safe pharmacotherapies are yet available.

Thyroid hormone (TH), including triiodothyronine (T3) and thyroxine (T4), play essential roles in the regulation of metabolism, energy homeostasis, and cardiovascular functions in mammals^6^. Hypothyroidism is associated with weight gain and dyslipidemia, whereas patients with hyperthyroidism exhibit elevated metabolic rate, weight loss, and improved hyperlipidemia^7^. Indeed, TH is among the oldest arsenal of anti-obesity therapies and has also been shown to reduce serum low-density lipoprotein cholesterol and alleviate type 2 diabetes and atherosclerosis^8,9^. However, the pharmacological dose of TH causes multiple deleterious effects, including tachycardia, heart attack, muscle wasting, and osteoporosis^10^. Early attempts to use TH for weight reduction even led to increased mortality^11^.

TH and its mimetics increase metabolic rates and energy expenditure in multiple organs^6^. In muscle, TH enhances the production of ATP and simultaneously stimulates futile cycles involving Na^+^/K^+^ ATPase and SERCA (sarcoplasmic/endoplasmic reticulum Ca^2+^ ATPase), thus leading to increased ATP consumption^12^. Additionally, TH plays an important role in promoting adaptive thermogenesis in brown adipose tissue (BAT) through its central action as well as its direct effect on brown adipocytes to induce the expression of uncoupling protein-1 (UCP1) during cold adaptation^13^. This mitochondrial inner membrane protein dissipates energy into heat by uncoupling oxidative phosphorylation from ATP production^14,15^. However, systemic administration of TH at pharmacological doses paradoxically decreases thermogenesis in BAT^16,17^. There is also a growing body of evidence that TH and its metabolites induce the biogenesis of beige adipocytes, an inducible type of multilocular brown-like adipocytes which play critical roles in maintaining energy homeostasis and regulating glucose and lipid metabolism in both rodents and humans^18–20^. In cultured white adipocytes, TH treatment induces mitochondrial biogenesis, oxygen consumption, and UCP1 expression^21,22^. Treatment of mice with the TH analog GC-1 leads to the induction of beige adipocytes in both subcutaneous WAT and visceral WAT^22^. However, systemic administration of TH does not increase adaptive thermogenesis and glucose consumption in WAT^16^. The contribution of adaptive thermogenesis in BAT and WAT to the beneficial effects of TH on the reduction of body weight and improvement of glucose and lipid metabolism remains unclear.

In light of the fact that systemic administration of the pharmacological dose of TH causes multiple deleterious effects on several non-adipose tissues and also impair thermogenesis in BAT, we investigated whether the selective delivery of T3 to adipose tissue is sufficient to mimic its metabolic benefits while minimizing its harmful activities in non-adipose tissues. To this end, we developed T3-encapsulated liposomal nanoparticles (PLT3), which were conjugated with an adipose tissue homing peptide (CKGGRAKDC). This peptide was identified by in vivo phage display as a ligand binding to prohibitin highly enriched in the vasculature of adipose tissue^23^, and has been successfully used as an adipose-homing peptide for selective delivery of cargos to adipose tissues^24–26^. PLT3 led to selective enrichment of T3 in adipose tissues while T3 concentration in non-adipose tissues, including the central nervous system, was barely detectable. Our results demonstrated that PLT3-mediated delivery of T3 to adipose tissue potently induced biogenesis and thermogenesis of beige adipocytes without any feedback inhibition of adrenergic input to WAT. In comparison with systemic administration of T3, PLT3 exhibited superior therapeutic effects on amelioration of obesity and its related metabolic complication with negligible side effects. Furthermore, we unexpectedly identified adipose tissue as an important target mediating the therapeutic benefits of TH in amelioration of hypercholesterolemia and atherosclerosis independent of its hepatic actions.

## Results

### Construction and characterization of adipose-targeted liposomal nanoparticles for delivery of T3 (PLT3)

**Fig. 1:**
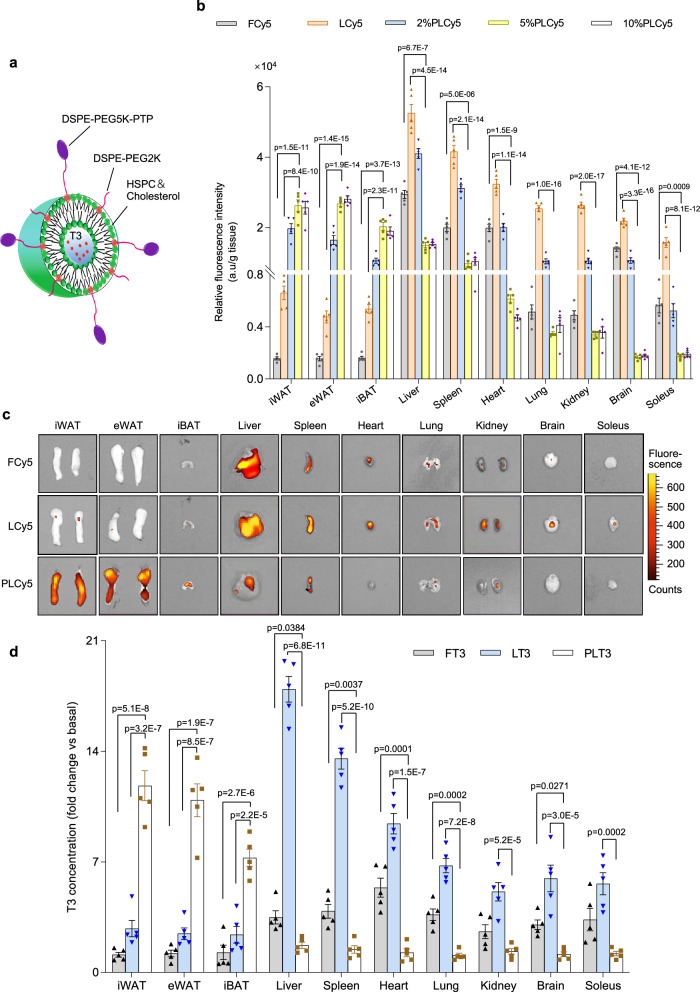
Construction and evaluation of liposomal nanoparticles for adipose-selective drug delivery. **a** Schematic diagram of the structure of triiodothyronine (T3)-encapsulated PTP-modified liposomes (PLT3), which is composed of HSPC, cholesterol, DSPE-PEG2K, DSPE-PEG5K-PTP, as well as T3. Cyanine 5 (Cy5) was encapsulated in the liposomes to trace the distribution of liposomes in vivo. **b**, **c** 8-week-old male C57BL/6 N mice were injected intraperitoneally (IP) with free Cy5 (FCy5), Cy5-encapsulated unmodified liposomes (LCy5), or Cy5-encapsulated liposomes modified with different molar ratios of PTP (PLCy5s) and sacrificed 8 h after injection. **b** Relative fluorescence intensities in inguinal white adipose tissue (iWAT), epididymal white adipose tissue (eWAT), interscapular brown adipose tissue (iBAT), liver, spleen, heart, lung, kidney, brain, and soleus were determined by a fluorimeter. **c** Ex vivo fluorescence imaging analysis for different tissues. **d** Fold changes of T3 concentration relative to saline-treated controls in different tissues of 8-week-old male C57BL/6 N mice were measured at 8 h after injection with FT3, LT3, PLT3, or saline. **b**–**d** *n* = 5 biologically independent replicates from one experiment. All data are expressed as mean ± SEM. One-way ANOVA followed by Least Significant Difference (LSD) test was applied for comparisons among multiple groups. Source data are available as a Source Data file.

T3 was encapsulated in liposomal nanoparticles modified with an adipose-homing peptide (PTP, with the amino acid sequence of CKGGRAKDC)^23^ to form PLT3 (Fig. 1a). To this end, the sulfhydryl group (-SH) of cysteine in PTP was covalently conjugated to the maleimide group in distearoyl-sn-glycero-3-phosphoethanolamine-N-[methoxy (polyethylene glycol)−5000-maleimide (DSPE-PEG5K-Mal) to synthesize DSPE-PEG5K-PTP. The successful chemical synthesis of DSPE-PEG5K-PTP was confirmed by the matrix-assisted laser desorption/ionization-time of flight (MALDI-ToF) spectrometry, showing that the synthetic product has a molecular weight (MW) of 6391 Da (Supplementary Fig. 1c), which was equal to the sum of PTP MW (1004 Da, Supplementary Fig. 1a) and DSPE-PEG5K-Mal MW (5387 Da, Supplementary Fig. 1b). Ultraviolet-visible (UV) spectrum analysis showed the disappearance of the absorption peak at 216 nm corresponding to the double bond of the maleimide group in the DSPE-PEG5K-PTP spectrum (Supplementary Fig. 1d). Likewise, proton nuclear magnetic resonance (^1^H-NMR) spectrum analysis confirmed that the signal peak (at 6.75 ppm) contributed by the methine proton of the maleimide group in DSPE-PEG5K-Mal was absent in the DSPE-PEG5K-PTP spectrum (Supplementary Fig. 1e). PLT3 nanoparticles with different molar ratios of PTP (2%, 5%, and 10% to total lipids) were constructed by assembling all the essential components, including hydrogenated soybean phosphatidylcholine (HSPC), cholesterol, distearoyl-sn-glycero-3-phosphoethanolamine-N-[methoxy (polyethylene glycol)−2000 (DSPE-PEG2K), DSPE-PEG5K-PTP, as well as T3 (Fig. 1a). The T3-encapsulated liposomes without the modification with PTP (LT3) possessed the same components as PLT3 except for replacing DSPE-PEG5K-PTP with DSPE-PEG5K-Mal. Sizes of PLT3s with different PTP ratios were similar to that of LT3, indicating that incorporation of PTP did not influence the size of the liposomes(Supplementary Fig. 1f). The zeta potential of liposomes was steadily elevated with the increasing amount of PTP (from −9.2 mV to +7.4 mV), due to the positive charge of PTP (Supplementary Fig. 1g). Electron microscopy imaging demonstrated homogeneous, well-defined spherical shape of PLT3 (Supplementary Fig. 1h). As expected, in vitro release analysis demonstrated that T3 encapsulated in LT3 and PLT3 liposomes was released in a much slower rate than in FT3 (Supplementary Fig. 1i). Approximately 43% and 65% of T3 was released from 5%PLT3 after 8 and 72 h respectively, whilst demulsification with acetonitrile led to a rapid and complete release of T3 from 5%PLT3, suggesting that lipid bilayers slowed down T3 release.

### PTP-modified liposomes are selectively enriched in adipose tissues

To validate the selective targeting capacity of PTP-modified liposomal nanoparticles to adipose tissues and to determine the optimal PTP concentration in liposomes and administration route, the fluorescent dye cyanine 5 (Cy5) was encapsulated in different concentrations of PTP-modified liposomes (PLCy5). Intraperitoneal (IP) injection of PLCy5 (5%PLCy5) led to a higher percentage of Cy5 accumulation in both inguinal subcutaneous WAT (iWAT) and epididymal WAT (eWAT) compared to subcutaneous and intravenous injection (Supplementary Fig. 2a). The highest fluorescence intensity in iWAT, eWAT, and interscapular brown adipose tissue (iBAT) was detected 8 h after IP injection (Supplementary Fig. 2b). Compared to free Cy5 (FCy5) and Cy5 encapsulated in unmodified liposmes (LCy5), mice injected with PLCy5 displayed a significantly higher level of fluorescence intensities in all three major adipose depots, including iWAT, eWAT, and iBAT (Fig. 1b, c). In contrast, retention of PLCy5 in non-adipose tissues, including liver, spleen, heart, lung, kidney, brain, and soleus, was remarkably reduced than that of LCy5 (Fig. 1b, c). Furthermore, mice injected with 5%PLCy5 exhibited significantly higher Cy5 accumulation in adipose tissues but much less Cy5 in other non-adipose tissues compared to LCy5 and 2%PLCy5, whereas adipose-targeting ability was comparable between 5%PLCy5 and 10%PLCy5 (Fig. 1b).

Consistent with the in vivo findings above, mouse adipocytes incubated with 5%PLCy5 exhibited significantly higher cellular uptake of fluorescent liposomes than cells incubated with 2%PLCy5 and LCy5, whereas there was no significant difference between 5%- and 10%PLCy5 groups (Supplementary Fig. 3a, b). On the other hand, cellular uptake of 5%PLCy5 was significantly decreased by preincubation of adipocytes with an excessive amount of PTP, suggesting that PTP facilitates liposomal uptakes in adipocytes. Likewise, treatment of mouse adipocytes with 5%- and 10%PLT3 led to the highest level of induction in the expression of browning markers including *Ucp1* and *Pgc1α* among all the T3 formulations (Supplementary Fig. 3c). Therefore, 5%PLT3 was chosen for further studies in mice. Analysis of T3 distribution with ELISA showed that T3 was preferentially enriched in the three adipose depots in PLT3-treated mice, whereas T3 was widely distributed in non-adipose tissues in FT3- and LT3-treated mice (Fig. 1d), demonstrating that PLT3 can selectively deliver T3 into adipose depots.

### PLT3 is much more effective than LT3 and FT3 in inducing weight loss

**Fig. 2:**
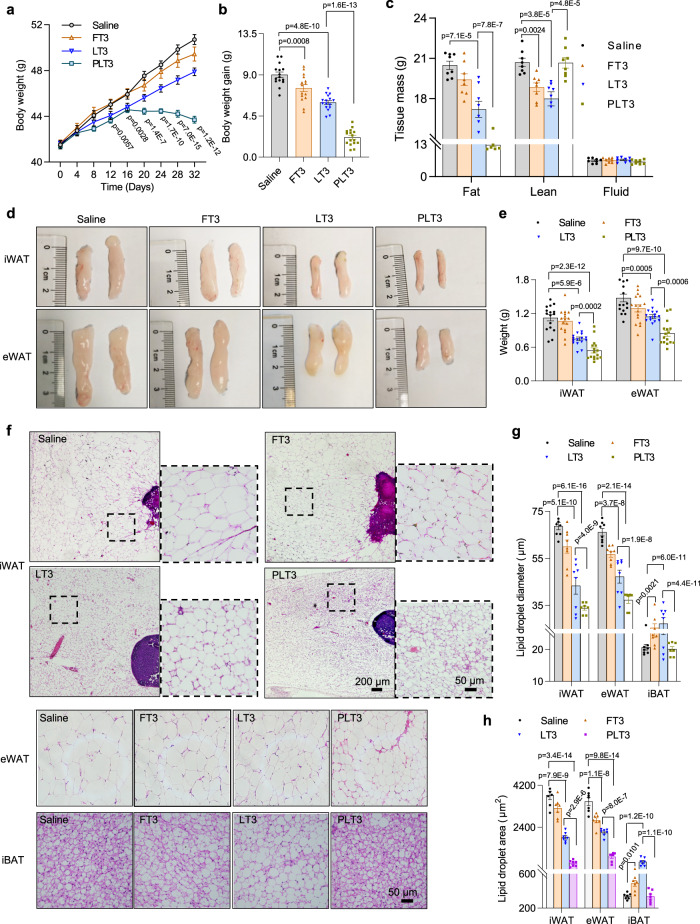
PLT3 is more efficient than FT3 and LT3 in counteracting obesity and reducing adiposity. 8-week-old male C57BL/6 N mice were fed with high-fat diet (HFD) for 8 weeks followed by IP injection with different forms of T3 or saline every 2 days for 32 days. **a** Dynamic changes in body weight during the treatment. The *p* values are for comparing each group to saline group. **b** Comparison of body weight gain among different groups from the 0^th^ day to the 32^th^ day. **c** Fat mass, lean mass, and body fluid of mice were measured by a Body Composition Analyzer. **d** Gross appearance and **e** wet weight of iWAT and eWAT. **f** Hematoxylin and eosin (H&E) staining of iWAT, eWAT, and iBAT. Graphs are from one experiment. **g** Diameter and **h** area of lipid droplets in iWAT, eWAT, and iBAT. **a**, **b**, **d**, **e** *n* = 15 (Saline and FT3) or 16 (LT3 and PLT3) biologically independent replicates from three independent experiments. **c** *n* = 8 biologically independent replicates from two independent experiments. **f**–**h** *n* = 7 (Saline and FT3) or 8 (LT3 and PLT3) biologically independent replicates from one experiment. All data are expressed as mean ± SEM. One-way ANOVA followed by LSD test was applied for comparisons among multiple groups. All the *p* values were two-sided. Source data are available as a Source Data file.

To compare the effects of different delivery forms of T3 on body weight and fat mass, male C57BL/6 N mice were fed with high-fat diet (HFD) for 8 weeks, followed by IP injection of FT3, LT3, or PLT3 at a T3 dose of 50 μg/kg body weight or saline control every two days for another 32 days. Blank PTP-decorated liposomes had no significant effects on body weight, fat mass, wet weight of iWAT and eWAT, whole-body oxygen consumption rate (VO_2_), glucose excursion, serum insulin, and cholesterol levels compared to saline-treated obese mice (Supplementary Fig 4). Therefore, saline-treated mice were used as a control for subsequent experiments. Mice receiving saline treatment for 32 days continued to gain weight from 41.7 ± 0.25 g to 50.71 ± 0.43 g (Fig. 2a). FT3- and LT3-treated mice exhibited 16.16% and 32.41% reduction in HFD-induced weight gain respectively, compared to saline-treated controls (Fig. 2b). Notably, the body weight gain of PLT3-treated mice was substantially decreased by 75.45% compared to mice receiving saline treatment (Fig. 2b). Likewise, PLT3-treated mice exhibited a much greater degree of reduction in fat mass than FT3- and LT3-treated mice (Fig. 2c). In contrast, FT3- and LT3-treated mice, but not PLT3-treated mice, displayed significant decrease in lean mass compared to saline control (Fig. 2c). Further analysis of individual adipose depots showed that the mass and weight of both iWAT and eWAT in PLT3-treated mice were significantly lower than those in FT3- and LT3-treated groups (Fig. 2d, e). Likewise, adipocyte sizes in both iWAT and eWAT of PLT3-treated mice were substantially smaller than those in the other two T3-treated groups (Fig. 2f–h), indicating that PLT3 is more effective in reducing lipid accumulation in white adipocytes. In contrast, lipid droplet size in iBAT was significantly increased in both FT3- and LT3-treated mice but remained unchanged in PLT3-treated mice (Fig. 2f–h).

### PLT3 obviates the suppressive effects of LT3 and FT3 on sympathetic activity and potently induces thermogenesis in WAT

**Fig. 3:**
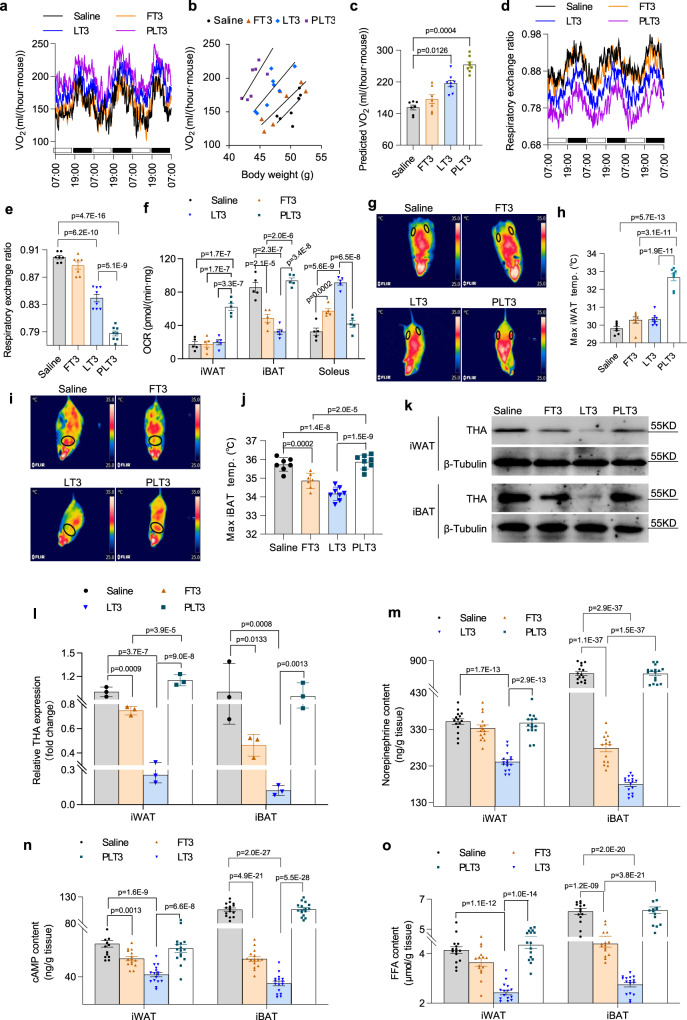
PLT3 potently stimulates energy expenditure and thermogenesis in WAT by avoiding feedback inhibition of adrenergic input to WAT. Male C57BL/6 N mice on HFD for 8 weeks were IP administered with different forms of T3 or saline for 32 days and then subjected to analysis with the Comprehensive Lab Animal Monitoring System (CLAMS). **a** Whole-body oxygen consumption rate (VO_2_) of mice during 72-h light/dark cycles. **b** Regression plots of VO_2_ against body weight. **c** ANCOVA-predicted whole-body VO_2_ at the mean body weight (50.14 g) of saline-treated mice. **d**, **e** Respiratory exchange ratio (RER) of mice. Blank and black boxes above the *X* axis represent light and dark phases respectively in (**a**) and (**d**). **f** Oxygen consumption rates (OCRs) in various tissues were measured by a Seahorse bioanalyzer. **g** Representative infrared thermography and **h** quantification of the skin temperature surrounding iWAT (black circles). **i** Representative infrared thermography and **j** quantification of the skin temperature surrounding iBAT (black circles). **k** The abundance of protein expression of throsine hydroxylase (THA) determined by western blot and **l** densitometry quantification of THA protein in iWAT and iBAT. **m** Norepinephrine content, **n** cyclic adenosine monophosphate (cAMP) content, and **o** free fatty acid (FFA) content in iWAT and iBAT. **a**–**e**, **g**–**j** *n* = 7 (Saline and FT3) or 8 (LT3 and PLT3) biologically independent replicates from one experiment. **f** *n* = 5 biologically independent replicates from one experiment. **k**, **l** *n* = 3 biologically independent replicates from one experiment. **m**–**o** *n* = 15 (Saline and FT3) or 16 (LT3 and PLT3) biologically independent replicates from three independent experiments. All data are expressed as mean ± SEM. One-way ANOVA followed by LSD test was applied for comparisons among multiple groups. All the *p* values were two-sided. Source data are available as a Source Data file.

The whole-body energy consumption as determined by oxygen consumption rate (VO_2_) and respiratory exchange ratio (RER) of mice during the treatments were determined using the Comprehensive Lab Analysis Monitoring System (CLAMS). At both the 14th and 28nd day after treatment, PLT3 was significantly more potent than LT3 and FT3 in increasing VO_2_ (Supplementary Fig. 5a, b & Fig. 3a–c), whereas there was no obvious difference in food intake and locomotor activity among different treatment groups (Supplementary Fig. 5c, d). Moreover, mice supplemented with PLT3 displayed the lowest RER, indicating that PLT3-treated mice preferentially consumed lipids as the energy source (Fig. 3d, e). Further examination of energy expenditure in individual metabolic tissues using a Seahorse bioanalyzer showed that the oxygen consumption rate (OCR) of iWAT in PLT3-treated mice was markedly elevated by 2.50 folds, compared to the saline group, while the OCR of iWAT depot remained unchanged in FT3- and LT3-treated mice (Fig. 3f). In contrast, FT3 and LT3, but not PLT3, significantly increased OCR in the liver (Supplementary Fig. 6a). Notably, whilst FT3 and LT3 modestly increased the gene expression of hepatic *fgf21* (Supplementary Fig. 6b) as well as circulating level of FGF21 (Supplementary Fig. 6c), PLT3 decreased hepatic *fgf21* mRNA expression and serum FGF21 concentration probably secondary to reduction in obesity^27^. In line with the changes in liver, FT3 and LT3 increased OCR in soleus muscle (Fig. 3f), accompanied by elevated expression of mitochondrial genes (*cytochrome c oxidase subunit 1* and *cytochrome c oxidase subunit 3)* and myosin heavy chain I, but decreased expression of myosin heavy chain IIA (Supplementary Fig. 6d, e), suggesting a switch from slow-twitch to fast-twitch fibers. On the other hand, PLT3 had no obvious effects in soleus muscle. Interestingly, FT3 and LT3 significantly decreased the OCR in iBAT (Fig. 3f). In line with the changes of OCRs, a significant elevation of the skin surface temperature in the inguinal region was observed only in PLT3-treated mice but not in FT3- or LT3-treated mice (Fig. 3g, h), suggesting that adipose-targeted delivery of T3 can increase the thermogenesis in iWAT. FT3- or LT3-treated mice displayed reduced skin surface temperature in the iBAT region. Only a modest elevation of skin surface temperature in the iBAT region was observed in PLT3-treated mice (Fig. 3i, j).

Given that adrenergic signals from sympathetic nerve system (SNS) plays a central role in controlling thermogenesis of adipose tissues^28^, we next investigated whether the differential effects of PLT3, LT3, and FT3 on thermogenesis in BAT and WAT are attributed to distinct changes in SNS. Immunoblotting analysis showed that the protein abundance of tyrosine hydroxylase, the rate-limiting enzyme in the biosynthesis of catecholamines, was significantly reduced in both iWAT and iBAT of FT3- and LT3-treated mice, whereas it remained unchanged in iWAT and iBAT of PLT3-treated mice (Fig. 3k, l and Supplementary 7a, b). Consistently, the content of norepinephrine (Fig. 3m), cyclic adenosine monophosphate (cAMP) (Fig. 3n), and free fatty acids (Fig. 3o) in both iWAT and iBAT was reduced in FT3- and LT3-treated mice but remained unaltered in the PLT3 group. Taken together, these findings suggest that adipose-targeted delivery of T3 by PLT3 can prevent the suppressive effects of systemic T3 administration by FT3 and LT3 on SNS activity in WAT and BAT, thereby promoting thermogenesis.

**Fig. 4:**
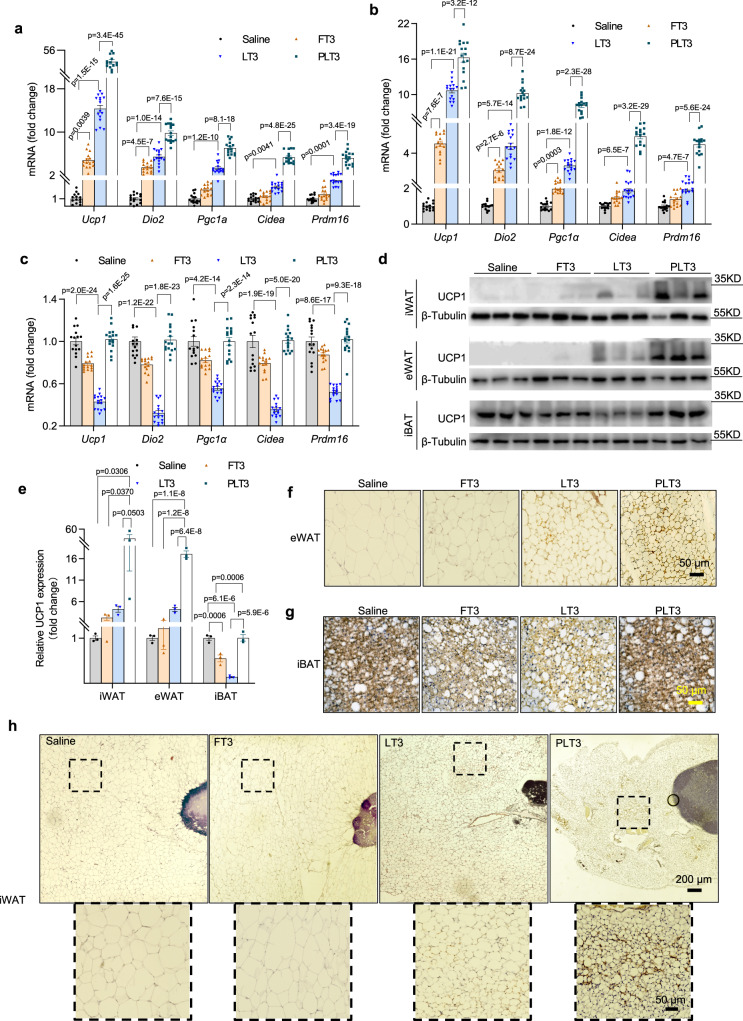
PLT3 is more potent than FT3 and LT3 to induce browning of WAT. HFD-fed C57BL/6 N mice were treated with different forms of T3 or saline for 32 days as in Fig. 2. **a**–**c** The mRNA levels of brown adipocyte markers in iWAT (**a**), eWAT (**b**), and iBAT (**c**) were determined by real-time PCR analysis. **d** The abundance of UCP1 protein expression was determined by western blot and **e** densitometry quantification of UCP1 protein in iWAT, eWAT, and iBAT. **f**–**h** Immunohistochemistry (IHC) staining of UCP1 in eWAT (**f**), iBAT (**g**), and iWAT (**h**). **a**–**c**, **f**–**h** *n* = 15 (Saline and FT3) or 16 (LT3 and PLT3) biologically independent replicates from three independent experiments. **d**, **e**, *n* = 3 biologically independent replicates from one experiment. All data are expressed as mean ± SEM. One-way ANOVA followed by LSD test was applied for comparisons among multiple groups. All the *p* values were two-sided. Source data are available as a Source Data file.

Compared to FT3 and LT3, PLT3 was much more potent in inducing the gene expressions of several browning markers (Fig. 4a, b) and in increasing UCP1 protein abundance (Fig. 4d, e & Supplementary 7c, d) and multilocular UCP1^+^ adipocytes in both iWAT (Fig. 4h) and eWAT (Fig. 4f). Notably, the mRNA levels of several browning markers, UCP1 protein abundance, and multilocular UCP1^+^ adipocytes in iBAT were significantly suppressed in mice supplemented with FT3 or LT3 in comparison with mice treated with saline (Fig. 4c–e, g & Supplementary 7e), but were modestly elevated or remained unchanged in iBAT of PLT3-treated mice (Fig. 4c–e, g & Supplementary 7e).

### PLT3 potently ameliorates obesity-induced adipose inflammation, glucose intolerance, insulin resistance, and fatty liver

**Fig. 5:**
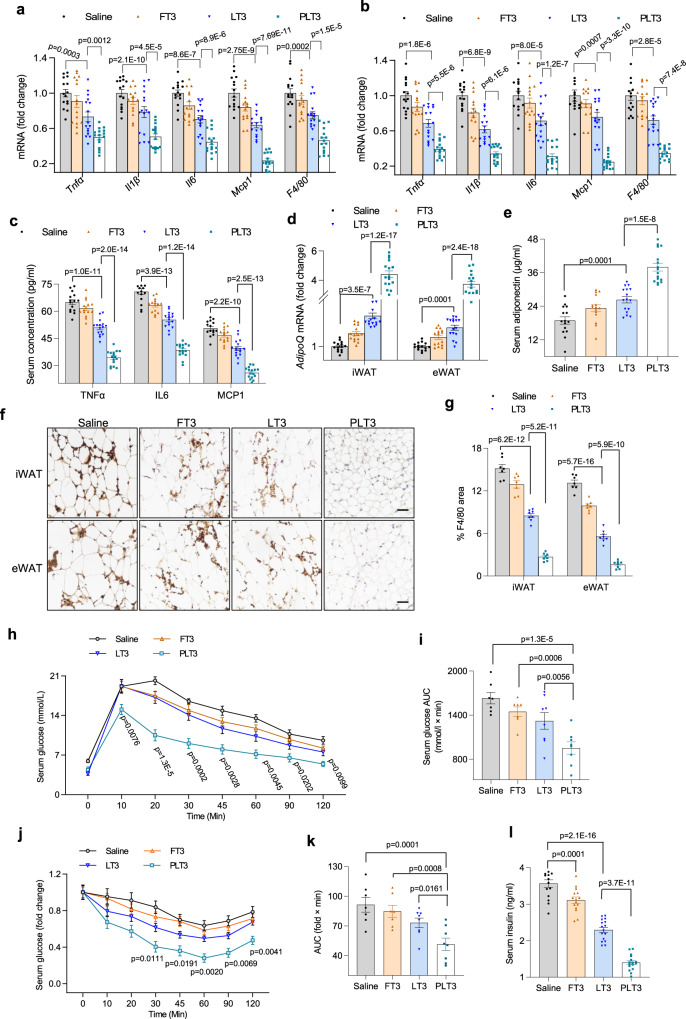
PLT3 is more effective than FT3 and LT3 in reducing obesity-evoked adipose inflammation, glucose intolerance, and hyperinsulinemia. HFD-induced male obese mice were IP administered with different forms of T3 or saline for 32 days. **a**, **b** The mRNA levels of proinflammatory factors in iWAT (**a**) and eWAT (**b**) determined by real-time PCR analysis. **c** Serum levels of several proinflammatory cytokines determined by ELISA. **d** The mRNA level of *adiponectin* in iWAT and eWAT. **e** Serum level of adiponectin measured by ELISA. **f** IHC staining and **g** densitometry quantification of F4/80 in iWAT and eWAT, scale bar: 50 μm. **h** Glucose excursion curve for IP glucose tolerance test (ipGTT). The *p* values are for comparing each group to saline group. **i** Area under the curve for ipGTT (**h**). **j** Glucose excursion curve for intraperitoneal insulin tolerance test (ipITT). The *p* values are for comparing each group to saline group. **k** Area under the curve for ipITT (**j**). **i** Fasting serum insulin level. **a**–**e**, **l** *n* = 15 (Saline and FT3) or 16 (LT3 and PLT3) biological replicates from three independent experiments. **f**–**k** *n* = 7 (Saline and FT3) or 8 (LT3 and PLT3) biological replicates from one experiment. All data are expressed as mean ± SEM. One-way ANOVA followed by LSD test was applied for comparisons among multiple groups. All the *p* values were two-sided. Source data are available as a Source Data file.

Given that PLT3 is more potent than LT3 and FT3 in inducing browning and thermogenesis in WAT, we further investigated the impacts of these three T3 delivery systems on inflammation and adipokine production, which are important contributors to obesity-related metabolic complications^29^. Real-time PCR analysis showed that PLT3 was much more effective than FT3 and LT3 in suppressing the gene expression of several inflammatory cytokines (*Tnfα*, *Il1β, Il6, Mcp1*) and the macrophage marker *F4/80* in both iWAT and eWAT of dietary obese mice (Fig. 5a, b). Furthermore, treatment with PLT3, but not FT3 or LT3, led to significant reduction in serum levels of TNFα, IL1β, and MCP1 (Fig. 5c), and marked elevation in the gene expression as well as the circulating concentration of adiponectin (Fig. 5d, e), which is an anti-inflammatory adipokine with insulin-sensitizing activities^30^. Likewise, PLT3 was much more potent than FT3 and LT3 in decreasing the number of infiltrated F4/80^+^ macrophages in iWAT and eWAT of obese mice (Fig. 5f, g). Furthermore, PLT3-treated mice exhibited significant improvements in glucose intolerance (Fig. 5h, i), insulin resistance (Fig. 5j, k), and hyperinsulinemia (5 l), suggesting that adipose-targeted delivery of T3 potently enhances insulin sensitivity.

**Fig. 6:**
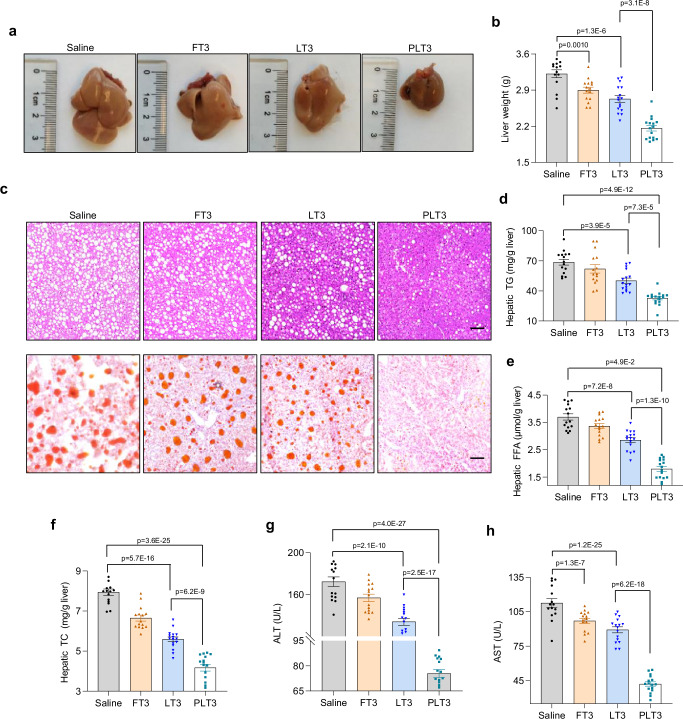
PLT3 is more potent than FT3 and LT3 in mitigating hepatosteatosis and liver injury. HFD-induced obese mice were administered with different forms of T3 or saline for 32 days as in Fig. 2. **a** Representative images of gross appearance and **b** wet weight of the liver. **c** H&E staining (upper panel) and Oil Red O staining (lower panel) of liver sections, scale bars: 100 μm (H&E staining) and 50 μm (Oil Red O staining). Graphs are from one experiment. **d** The content of triglyceride (TG), **e** free fatty acid (FFA), and **f** total cholesterol (TC) in the liver was quantified by biochemical assays. **g**, **h** Serum levels of alanine aminotransferase (ALT) activity (**g**) and aspartate aminotransferase (AST) activity (**h**). All data are expressed as mean ± SEM. *n* = 15 (Saline and FT3) or 16 (LT3 and PLT3) biologically independent replicates from three experiments. One-way ANOVA followed by LSD test was applied for comparisons among multiple groups. All the *p* values were two-sided. Source data are available as a Source Data file.

Although the weight and size of the livers were modestly decreased in FT3- and LT3-treated mice, the magnitude of reduction in PLT3-treated mice was much more pronounced than the other two T3-treated groups (Fig. 6a, b). Consistently, both H&E and Oil Red-O staining of liver sections revealed that PLT3 led to a substantially greater degree of reduction in lipid deposition than FT3 and LT3 (Fig. 6c). These changes in PLT3-treated mice were mirrored by more obvious decrease in hepatic levels of triglyceride (TG), free fatty acid (FFA), and total cholesterol (TC) (Fig. 6d–f), as well as in serum levels of the liver injury markers, including alanine aminotransferase (ALT) and aspartate aminotransferase (AST) (Fig. 6g, h).

To determine if PLT3 exerts its metabolic benefits in adipose tissues independent of weight loss, we performed metabolic profiling analysis at day 7 after treatment, a time point when body weight remains unchanged (Supplementary Fig. 8a). Treatment with PLT3 for 7 days led to significant improvement in glucose intolerance (Supplementary Fig. 8b,c), hyperinsulinemia (Supplementary Fig. 8d), hepatic steatosis (Supplementary Fig. 8e), accompanied by an elevation in circulating adiponectin (Supplementary Fig. 8f), whereas treatment with LT3 and FT3 had no obvious effect (Supplementary Fig. 8b–f) suggesting that the metabolic benefits of PLT3 are not merely attributed to the reduction in obesity.

### PLT3 is more effective than FT3 and LT3 in reducing hypercholesterolemia and protecting against atherosclerosis

**Fig. 7:**
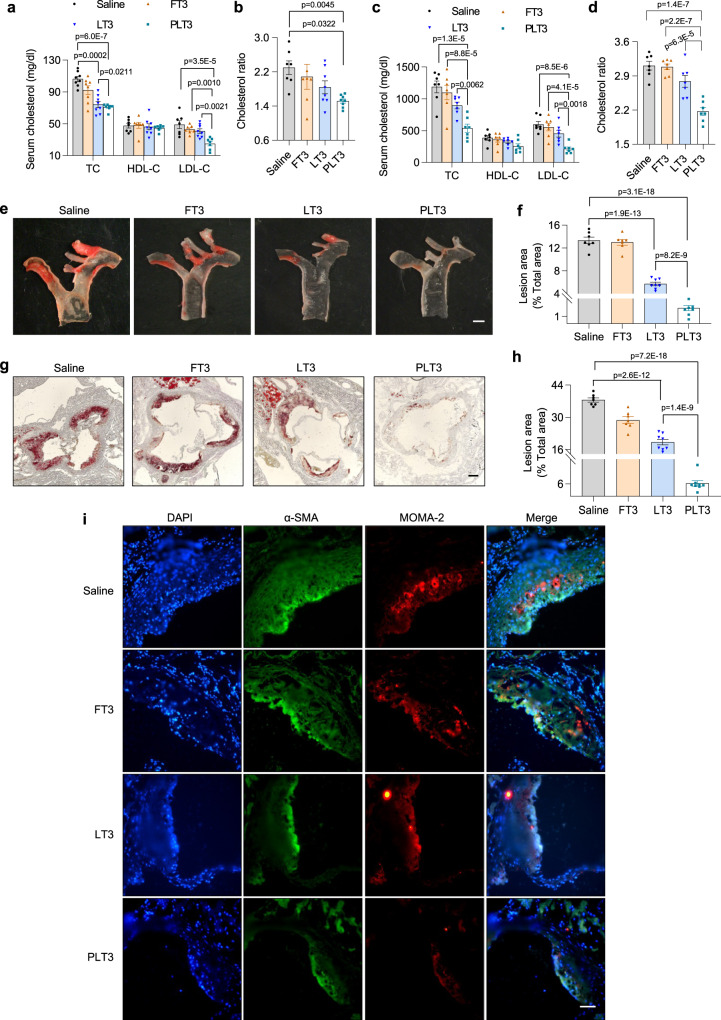
PLT3 is more potent than FT3 and LT3 in reducing hypercholesterolemia and protecting against atherosclerosis. **a** Serum levels of total cholesterol (TC), high-density lipoprotein cholesterol (HDL-C), and low-density lipoprotein cholesterol (LDL-C), and **b** cholesterol ratio (TC/HDL-C) in HFD-fed C57BL/6 N mice treated with different forms of T3 or saline for 32 days as in Fig. 2. **c**–**i** ApoE^−/−^ mice on HFHCD were IP injected with different forms of T3 or saline for 2 months. **c** Serum levels of TC, HDL-C, and LDL-C, and **d** cholesterol ratio in apoE^−/−^ mice. **e** Representative photographs of aortas in the en face preparation stained with Oil Red O and **f** quantification of atherosclerotic area, scale bar: 1 mm. **g** Representative histological images of the aortic sinus stained with oil red O and **h** quantification of atherosclerotic area, scale bar: 100 μm. **i** Immunofluorescence staining of α-smooth muscle actin (α-SMA) and monocyte/macrophage-2 (MOMA-2) in the atherosclerotic lesion area of the aortic sinus, scale bar: 50 μm. **a**, **b**, **e**–**i**, *n* = 7 (Saline and FT3) or 8 (LT3 and PLT3) biological replicates from one experiment. **c**, **d** *n* = 7 biological replicates from one experiment. All data are expressed as mean ± SEM. One-way ANOVA followed by LSD test was applied for comparisons among multiple groups. All the *p* values were two-sided. Source data are available as a Source Data file.

TH and its mimetics effectively lower cholesterol levels and therefore have anti-atherosclerotic potentials^8,31^. Therefore, we comparatively evaluated the effects of systemic and adipose-targeted delivery of T3 on serum lipid profiles and atherogenesis. In both HFD-induced obese mice and high-fat high-cholesterol diet (HFHCD)-fed induced hypercholesterolemic apoE^−/−^ mice, FT3 and LT3 modestly decreased serum levels of total cholesterol (TC) and low-density lipoprotein cholesterol (LDL-C), as well as cholesterol ratio (TC/high-density lipoprotein cholesterol), the latter which of is regarded as a better indicator for heart disease (Fig. 7a–d). Intriguingly, the magnitude of reduction in TC, LDL-C, and cholesterol ratio in PLT3-treated mice was significantly greater than that in FT3- and LT3-treated mice (Fig. 7a–d), suggesting that T3 can lower cholesterol through its actions in adipose tissues.

In line with the changes in serum lipid profiles, PLT3 was much more potent than LT3 in reducing the size of aortic atheromatous plaques, as determined by Oil Red O staining of the aorta arch, whereas FT3 had no significant effects compared to saline control (Fig. 7e, f). Likewise, histologic analysis of the aortic roots showed that PLT3 group displayed much more pronounced reductions in areas of the lipid-laden atherosclerotic lesion (Fig. 7g, h), smooth muscle cell proliferation, and macrophage infiltration than FT3 and LT3 groups (Fig. 7i). Surprisingly, the effects of PLT3 on reduction of TC and LDL-C occurred as early as 5 days after PLT3 treatment, independent of body weight change (Supplementary Table 1).

### The metabolic benefits of PLT3 are dependent on adipose tissue

To further confirm that PLT3 exerts its metabolic benefits by targeting adipose tissues, we evaluated the effects of PLT3 treatment in a mouse model with lipodystrophy previously established in our laboratory by adipocyte-selective ablation of murine double minute 2 (MDM2), which exhibits a complete loss of all adipose tissues, accompanied with severe hyperglycemia, hyperinsulinemia, hypercholesterolemia, and fatty liver^32^. Treatment of lipodystrophic mice with the same dose of PLT3 as used in dietary obese mice had no significant effects on body weight, whole-body energy expenditure, glucose intolerance, serum insulin, fatty liver, and serum TC as compared to vehicle-treated mice (Supplementary Fig. 9), suggesting that adipose tissue is obligatory for mediating the systemic effects of PLT3 on glucose/lipid metabolism and insulin sensitivity.

To interrogate whether PLT3-induced thermogenesis in WAT and reduction in fat mass are mediated by induction of UCP1, we compared the effects of PLT3 between HFD-fed UCP1 knockout (KO) mice and wildtype (WT) littermates. HFD-fed UCP1 KO mice treated with PLT3 also exhibited significantly decreased body weight and fat mass compared to those treated with vehicle control (Supplementary Fig. 10a–d). However, the effects of PLT3-induced reduction in body weight gain and fat mass in UCP1 KO mice were significantly lower than those in WT littermates (Supplementary Fig. 10b–d). In both types of mice, PLT3 treatment had no obvious effects on lean mass and body fluid (supplementary Fig. 10e, f). Likewise, treatment with PLT3 was still able to increase whole-body energy expenditure and OCR in iWAT in UCP1 KO mice, whereas such effects of PLT3 in UCP1 KO mice were significantly weaker than those in WT littermates (Supplementary Fig. 10g–i), suggesting that PLT3-induced thermogenesis in WAT is possibly mediated by both UCP1-dependent and UCP1-independent unknown mechanisms which require further characterization.

### TH-evoked cardiac toxicity is absent in mice treated with PLT3

**Fig. 8:**
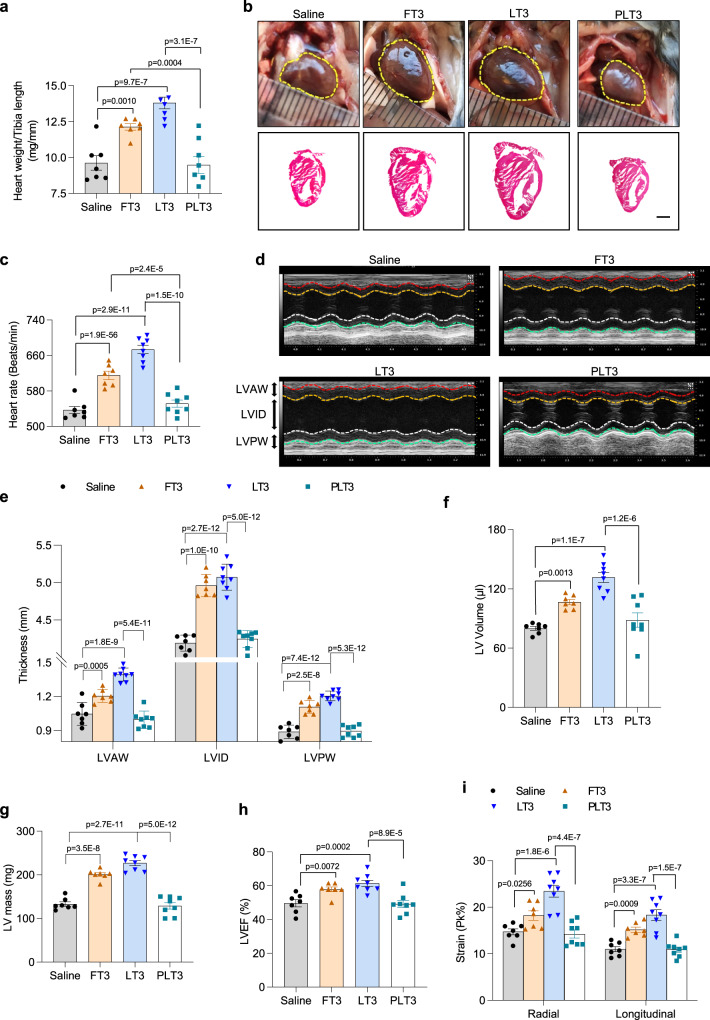
Mice treated with PLT3 are devoid of cardiac toxicity. The morphology and functions of hearts in male C57BL/6 N mice after 32 days of supplementation with FT3, LT3, PLT3, or saline were measured. **a** The ratio of heart weight to tibia length. **b** Representative images of gross appearance (upper panel) and H&E-stained cross-sections of the hearts (lower panel). Graphs are from one experiment, scale bar: 2 mm. **c** Heart rate of mice. **d** Representative M-mode echocardiographic images of left ventricular (LV) short-axis, LVAW: LV anterior wall thickness, LVID: LV inner dimension, LVPW: LV posterior wall thickness. Quantification of **e** end-diastolic LV wall thicknesses, **f** LV volume, **g** LV mass, **h** LV ejection fraction (LVEF), and **i** cardial strains of the hearts. All data are expressed as mean ± SEM. *n* = 7 (Saline and FT3) or 8 (LT3 and PLT3) biological replicates from one experiment. One-way ANOVA followed by LSD test was applied for comparisons among multiple groups. All the *p* values were two-sided. Source data are available as a Source Data file.

Cardiotoxicity is one of the most severe concerns for TH pharmacotherapy^33^. Indeed, despite the relatively low dose of T3 used, both the weight and size of mouse hearts were obviously increased by FT3 and LT3, but not affected by PLT3 (Fig. 8a, b). Likewise, the heart rates in FT3- and LT3-treated mice were augmented by 14.4% and 25.3% respectively, but remained unchanged in PLT3-treated mice compared to saline groups (Fig. 8c). Echocardiographic analysis showed that FT3 and LT3 significantly elevated left ventricular end-diastolic anterior wall thickness (LVAW), left ventricular end-diastolic inner dimension (LVID), left ventricular end-diastolic posterior wall thickness (LVPW), left ventricle (LV) volume, LV mass, and LV ejection fraction (LVEF). In contrast, these pathological changes in the heart were not observed in PLT3-treated mice (Fig. 8d–h). In addition, FT3- and LT3-treated mice, but not PLT3-treated mice, exhibited increased radial strain and longitudinal strain (Fig. 8i).

### PLT3 does not cause bone loss or disrupt neuroendocrine circuit

**Fig. 9:**
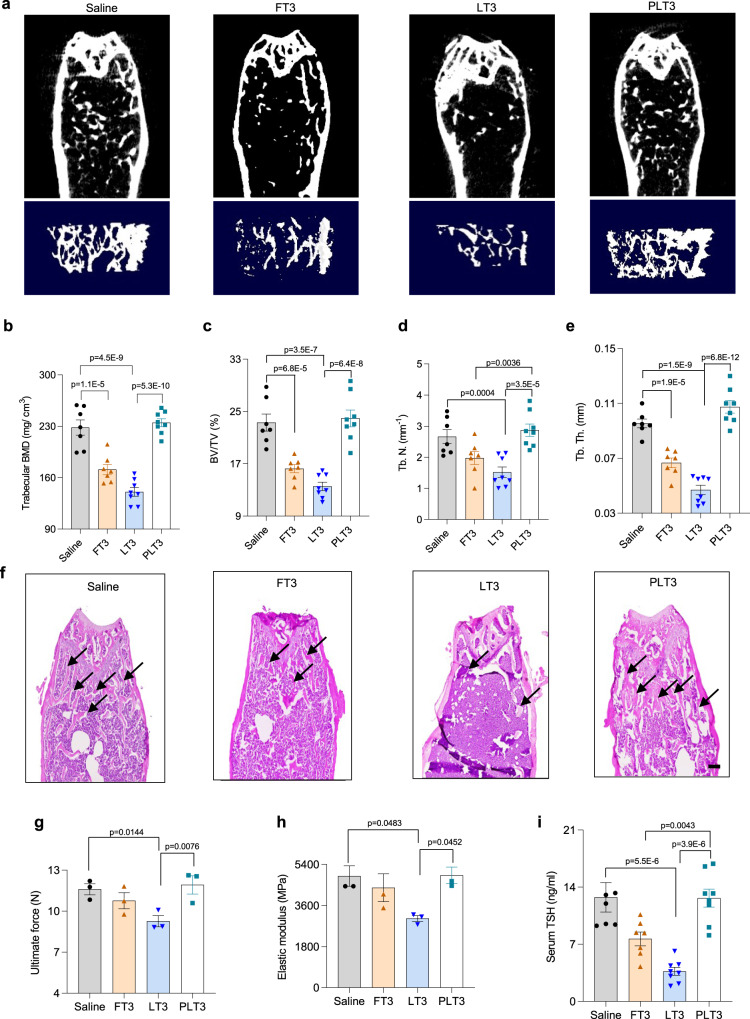
PLT3 does not cause osteoporosis or impair TSH secretion in mice. Male C57BL/6 N mice were treated with different forms of T3 or saline for 32 days as in Fig. 2. **a** Representative micro-CT images of distal femur (upper panel) and trabecular bone (lower panel). Graphs are from one experiment. **b** Trabecular bone mineral density (BMD), **c** the ratio of bone volume to total volume (BV/TV), **d** trabecular number (Tb. N), and **e** trabecular thickness (Tb. Th) at the distal femur. **f** H&E staining of femur bone. The black arrows represent trabecular bone. Scale bar: 200 µm. Graphs are from one experiment. **g**, **h** Three-point bending test for the femurs. Femurs were placed on the posterior surface across two supporting bars and a load was applied to the anterior surface of shaft until the bone was fractured to measure ultimate force (**g**) and elastic modulus (**h**). **i** The serum level of TSH was measured by ELISA. **a**–**f**, **i** *n* = 7 (Saline and FT3) or 8 (LT3 and PLT3) biological replicates from one experiment. **g**, **h** *n* = 3 biological replicates from one experiment. All data are expressed as mean ± SEM. One-way ANOVA followed by LSD test was applied for comparisons among multiple groups. All the *p* values were two-sided. Source data are available as a Source Data file.

Systemic administration of TH accelerates bone turnover and causes osteoporosis in rodents and humans^34^. We thus sought to investigate whether this deleterious effect can be minimized by adipose-selective delivery of T3. To this end, micro-computed tomography (Micro-CT) was performed on the femur of mice after 32 days of treatments with either saline or different forms of T3. The scanning images showed a dramatic bone loss in the distal femur and the trabecular bone of FT3- and LT3-treated mice, whereas the bone mineral density (BMD) of PLT3-treated mice was comparable to control mice (Fig. 9a, b). Further analysis demonstrated that FT3 and LT3 led to a reduction in bone volume (BV) fraction, trabecular number (Tb. N), and trabecular thickness (Tb. Th), indicative of severe disruption of bone structure (Fig. 9c–e). In contrast, these parameters were not altered in PLT3-treated mice, suggesting that adipose-targeted delivery of T3 does not cause bone loss. Consistently, H&E staining analysis also showed that trabecular number in femur was significantly decreased in both FT3- and LT3-treated mice, but remained unchanged in PLT3-treated mice (Fig. 9f). Furthermore, femurs from FT3- and LT3-treated, but not PLT3-treated mice exhibited significantly reduced ultimate force (Fig. 9g) and elastic modulus (Fig. 9h), as determined by three-point bending tests. The production of endogenous TH is tightly regulated by the hypothalamic-pituitary-thyroid axis, which is perturbed by overdose of exogenous TH. Indeed, thyroid-stimulating hormone (TSH) level was significantly suppressed by FT3 and LT3, whereas this effect was absent in PLT3-treated mice (Fig. 9i), demonstrating that the neuroendocrine circuit is not disturbed by adipose-selective delivery of T3.

## Discussion

TH is a potent endogenous inducer of energy expenditure and has therapeutic potentials for obesity, type 2 diabetes, and atherosclerosis^8–10^. However, due to the ubiquitous expression of TH receptors, the pharmacological dose of systemic TH therapy causes a host of severe side effects on non-adipose tissues, thereby limiting its therapeutic application^35–37^. Moreover, the weight loss effect of systemic TH administration is mild and unsustainable in both humans and animals, perhaps due to its complex central actions which compromises the thermogenic activity of TH^38^. In this paper, we developed T3-encapsulated liposomal nanoparticles (PLT3), which were decorated with an adipose-homing peptide^39^. PLT3 is more potent and sustainable than systemic T3 therapy in reducing obesity and a cluster of metabolic and vascular complications. Importantly, adipose-targeted delivery of T3 can uncouple its therapeutic benefits from multiple deleterious effects of systemic T3 therapy, including heart dysfunction, bone loss, muscle wasting, and perturbation of the neuroendocrine circuit. (Supplementary Fig. 11).

Skeletal muscle and adipose tissues are the two well-established major organs mediating the effects of TH on the stimulation of metabolic rate and energy expenditure. However, the muscular actions of TH are associated with severe muscle wasting^10^. During cold adaptation, TH is required for both BAT thermogenesis and browning of WAT through overlapping but distinct mechanisms^13^. TH receptor β (TRβ) is obligatory for T3-induced browning of WAT, but is dispensable for T3-evoked mitochondrial UCP1 expression and thermogenesis in BAT. Notably, chronic administration of a synthetic, TRβ-specific agonist (GC-1) to *ob/ob* mice induces marked browning of inguinal WAT (iWAT), but reduces UCP1 expression and thermogenic activity in BAT^40^, highlighting the complexity of systemic TH actions in adaptive thermogenesis in different adipose tissues. Indeed, our present study also found that systemic administration of T3 with LT3 or FT3 leads to impaired BAT activity but no change in thermogenic activity in WAT despite increased expression of UCP1. The impaired BAT activity by pharmacological doses of systemic TH has been observed in many previous studies^17,22,41^. Our findings suggest that the “paradoxical” reduction or no effect in thermogenic activity of BAT and WAT in mice receiving systemic T3 treatment is attributed to the suppression of SNS, as evidenced by reduced expression of tyrosine hydroxylase, decreased contents of norepinephrine, cAMP, and FFA, and attenuated lipolysis in adipose tissues of LT3 and FT3-treated mice. Given that systemic administration of T3 acts on muscle to generate excessive heat, we speculate that heat generated from these non-adipose tissues may act in a feedback manner to suppress SNS in adipose tissues by raising body temperature (Supplementary Fig. 11). In this connection, adipose-targeted T3 delivery with PLT3 is able to avoid such a disruption of the neuroendocrine circuit, thereby inducing marked elevations in UCP1 expression, oxygen consumption as well as thermogenesis in WAT. Furthermore, our findings suggest that TH-induced browning and thermogenesis of WAT alone is sufficient to induce whole-body energy expenditure and fat mass loss. In support of this notion, several previous studies have shown that browning of WAT can compensate for the functional loss of classical BAT in maintaining systemic energy homeostasis and preventing obesity^38^. It is worthy to note that we also observed a marked elevation of UCP1 in eWAT of PLT3-treated mice. Although it is well established that eWAT is resistant to browning and express much less UCP1 in response to cold exposure, administration of thermogenic agents such as the β3-adrenergic receptor agonists CL316,243 and norepinephrine strongly induced UCP1 expression in eWAT in both gene and protein levels^42–44^, suggesting that pharmacological agents such as CL316,243 and TH can also promote thermogenic activity of eWAT in mice.

In addition to its striking effects on browning and thermogenesis of WAT, our study observed that obese WAT was dramatically remodeled to a metabolically healthy phenotype, including marked reduction in macrophage infiltration and proinflammatory cytokines expression, but increased adiponectin level, an adipokine with anti-inflammatory and insulin-sensitizing activities^45,46^. These changes in WAT are not obvious in mice with systemic T3 therapy, and are possibly due to the indirect consequences of reduced adipocyte hypertrophy and/or increased beige adipocytes. Adipose inflammation, aberrant production of adipokines/cytokine, and hypoadiponectinemia are the well-known mediators linking obesity and its metabolic complications^47,48^. Therefore, reversal of these obesity-induced pathological abnormalities in WAT is likely to be the key contributor to the amelioration of glucose intolerance and insulin resistance in obese mice receiving adipose-targeted T3 therapy. Additionally, increased biogenesis of beige adipocytes, which possess potent activity in glucose uptake and disposal, may also lead to improved glucose homeostasis in PLT3-treated mice. In line with this notion, cold-induced browning of WAT is closely associated with significant amelioration of glucose dysregulation and insulin resistance in patients with type 2 diabetes (44, 47). A more comprehensive characterization of the WAT secretomes remodeled by PLT3 shall be carried out in the future to provide more insight on the humoral changes brought by PLT3.

An unexpected finding in our study is that adipose-targeted T3 therapy is much more potent than systemic T3 administration in reducing hypercholesterolemia, fatty liver, and atherosclerosis, independent of body weight reduction. The hepatic actions of TH are traditionally thought to be the primary mechanism responsible for its lipid-lowering activity, where TH acts through TRβ to induce the expression of LDL receptor, thereby facilitating hepatic uptake and clearance of LDL-C from the bloodstream^8^. However, TH remains active in decreasing hypercholesterolemia in LDLR-deficient mice^49^, suggesting the existence of additional hepatocyte-independent mechanism(s). Our present study showed that adipose-targeted T3 therapy retains the ability to reduce hypercholesterolemia in apoE^−/−^ substantially mice with a defect in hepatic uptake of LDL-C, whereas LT3 treatment, which causes preferential accumulation of T3 in the liver, leads to only a relatively modest reduction in circulating cholesterol in apoE^−/−^ mice, indicating that adipose tissue is another vital action site mediating the cholesterol-lowering activity of TH. In fact, WAT is a major site for cholesterol storage, accounting for over 50% of total body cholesterol in obesity^50^. Activation of thermogenic adipocytes by cold exposure or β3-adrenergic agonists has been shown to decrease circulating LDL-C, promote HDL turnover, and reverse cholesterol transport, thereby alleviating atherosclerosis^51,52^. Moreover, adipocyte-secreted adiponectin improves lipid profiles through its endocrine actions on the liver to suppress hepatic cholesterol biosynthesis and induce lipid β-oxidation, and also exerts anti-atherosclerotic activity through its direct effects on the vasculature for prevention of endothelial apoptosis and activation, suppression of foam cell formation as well as proliferation and migration of smooth muscle cells^53,54^. Thus, the potent activity of PLT3 in lowering cholesterol and protecting from atherosclerosis and hepatic steatosis is likely to be mediated by elevated adipocytes beiging, increased adiponectin level, and reduced proinflammatory cytokines or altered secretion of other unknown adipokines.

Over the past decade, many TH mimetics with improved safety profiles, including TRβ-selective agonists and liver-selective TH analogs, have been developed as potential therapeutics for obesity, diabetes, and atherosclerosis^8^. However, none of these TH analogs has been approved for clinical use so far because their adverse effects on heart, muscle, and neuroendocrine circuit remain problematic. Thus, our present study provides an alternative strategy to develop TH therapies for a cluster of obesity-related metabolic and vascular complications with superior efficacy and safety by releasing TH or activating TH receptors selectively in adipose tissues.

Apart from PTP-decorated liposomal delivery system used in our study, aptamers specific to adipocytes have been selected for potential adipose-targeted delivery^55,56^. Adipocyte-targeting aptamer-modified gold nanoclusters, resveratrol-loaded liposomes, and lipid nanocarriers have been used for inducing browning of 3T3-L1 white adipocytes^57^. However, the tissue specificity and efficiency of these delivery methods to adipose tissues have not been tested in vivo. Among different nanoparticles-based delivery system, liposomes remain the most popular choice due to their flexible physicochemical and biophysical properties, easy manipulation for encapsulation and surface modification, and excellent biocompatibility^58^, and have been clinically implemented for delivery of drugs to treat cancer^59^, severe fungal infection^60^, and analgesia^61^.

There are limitations in our study. Firstly, only the mouse models were used to test the therapeutic effects of PLT3. Due to the differences in anatomical locations and functions of adipose tissues between mice and humans, the translational potential of PLT3 needs to be further evaluated in large animals such as non-human primates before clinical evaluation. Secondly, PLT3 was administered through IP injection, which may not be convenient for patients. Non-invasive administration routes, such as microneedle-mediated transdermal delivery, shall be explored. Thirdly, as obesity-related cardiometabolic complications are chronic disorders, the effects of long-term PLT3 administration on obesity-related metabolic complications need to be tested in different models. Finally, given that cold exposure and pharmacological agents-induced browning of WAT is a reversible process^62,63^, further studies are required to evaluate the sustainability of PLT3-induced browning of WAT and weight loss after termination of the treatment.

## Methods

### Synthesis of DSPE-PEG5K-PTP

The -SH of cysteine in PTP (Synthesized by Genscript) was covalently conjugated to the maleimide group of DSPE-PEG5K-Mal (Avanti lipids, catalog no. 880224). PTP and DSPE-PEG5K-Mal were dissolved in separate portions of phosphate buffer (0.01 M, pH 8.0) and mixed at a molar ratio of 1.2: 1 (PTP: DSPE-PEG5K-Mal). The mixture was incubated in a water bath at 30 °C with continuous stirring. After 24 h, the synthetic product was purified by dialysis (Thermo, catalog no. 68100, MWCO: 3500 Da) against distilled water and freeze-dried. The molar weight of the product was verified with a MALDI-ToF spectrometer (Bruker, model rapifleX). For structural analysis, the ^1^H-NMR spectrum of product dissolved in CDCl_3_ was recorded on a Bruker spectrometer (Model DX 500) at 400 Hz. The UV spectrum of the product was acquired using a microreader (Molecular Devices, model SpectraMax M4).

### Preparation of liposomal nanoparticles

PLT3 nanoparticles comprised of HSPC (Avanti lipids, catalog no. 840058), cholesterol (Avanti lipids, catalog no. 700100), DSEP-PEG2K (Avanti lipids, catalog no. 880234, 2 mol% of total lipids), and DSPE-PEG5K-PTP (2, 5, or 10 mol% of total lipids) were prepared by a reverse-phase evaporation method. The lipids were dissolved in chloroform, and T3 was dissolved in phosphate-buffered saline (PBS, 0.01 M, pH 7.4). The organic solution was subsequently added to PBS at a volume ratio of 3: 1 (chloroform: PBS). The mixture was sonicated for 30 min to form a W/O emulsion and then placed on a rotary evaporator (BUCHI, model R-300) to obtain crude liposomes by removing the organic solvent. To reduce the size, crude liposomes were extruded against 400 nm (Whatman, catalog no. 110607), 200 nm (Whatman, catalog no. 110606), and 100 nm (Whatman, catalog no. 110605) filter successively with an extruder (Avestin, catalog no. LF-50).

### Characterization of liposomes

100 μL of the liposomes were diluted with 900 μL of PBS (0.01 M, pH 7.4). After vortex, particle size and zeta potential of the liposomes were determined by a dynamic light scattering instrument (Malvern, model Zetasizer Nano ZS).

The in vitro release of T3 from liposomes was performed using a dialysis method. The liposomal nanoparticles solution (0.2 mL) was transferred in a dialysis bag (Thermo, catalog no. 68100, MWCO: 3500 Da) and put in 20 mL fresh PBS solution (0.01 M, pH 7.4) containing 0.1% (v/v) Tween 80 in a flask, which was placed in a shaking incubator at 37 °C. At each time point, 0.5 mL of the sample was withdrawn and replaced with fresh release medium. T3 concentrations in the samples were determined by high-performance liquid chromatography (Agilent, catalog no. 1260). The cumulative percentage drug release (Er) was calculated as follows:

1 $${Er}\,(\%)=\frac{{{{{\rm{V}}}}}_{{{{\rm{e}}}}}{\sum }_{1}^{n-1}{C}_{i}+{{{{\rm{V}}}}}_{0}{{{{\rm{C}}}}}_{{{{\rm{n}}}}}}{{{{{\rm{M}}}}}_{{{{\rm{T}}}}3}}\times 100$$

V_e_ represents the volume of release medium removed every time (0.5 mL), C_*n*_ represents the concentration of T3 at nth sampling interval, ${\sum }_{1}^{n-1}{{{C}}}_{{{{\rm{i}}}}}$ represents the sum of concentrations T3 determined at sampling 1 intervals *n*–1, V_0_ represents the total volume of release medium (20 mL), M_T3_ represents the amount of T3 in the liposomal nanoparticles.

### Animal experiments

All the animal experiments were approved by the Institutional Animal Care and Use Committee at The University of Hong Kong. The Committee of the Use of Live Animals in Teaching and Research (CULATR) NO. is 4848-18. 8-week-old C57BL/6 N mice were obtained from the laboratory animal unit, University of Hong Kong. Mice were housed in a room under a controlled temperature (23 ± 1 °C) with free access to water and food. The sample sizes for mice were determined based on the mean value and standard deviation of body weight of HFD-induced obese mice in our previous studies^27,64,65^ to achieve statistical significance of *p* < 0.05 with 80% probability. According to our calculation, the minimal number of mice required for each group is 6. Therefore, we chose *n* = 8 for each group to ensure the statistical power. C57BL/6 N mice were fed with HFD (Research diets, catalog no. D12451) for 8 weeks and then randomized into each group according to body weight. Mice resistant to HFD-induced body weight gain have excluded those mice prior to initiation of the treatments. Obese mice were IP injected with FT3, LT3, or PLT3 at a T3 dose of 50 μg/kg or saline control every other day for 32 days. All T3 formulations were diluted to a concentration of 20 μg/ml and injected to mice at a volume of 2.5 ml/kg body weight. After treatment, body weight, fat mass, and lean mass were measured with a Body Composition Analyzer (Bruker, model LF90). Glucose tolerance test was conducted in overnight-fasted (19:00 pm−9:00am) mice by intraperitoneally injecting D-glucose (Sigma-Aldrich, catalog no. G5767) at a dose of 2 g/kg body weight. Insulin tolerance test was performed by intraperitoneally injecting insulin at a dose of 1 U/kg body weight to 6h-fasted (8:00am–14:00 pm) mice. The blood was collected from tail vein at various time points for measurement of glucose levels with an Accu-Check blood glucose meter. The surface temperature surrounding iWAT was recorded with an infrared camera (FLIR, model T440) and quantified using FLIR analysis software. At the end of the treatment, the mice were sacrificed to collect various tissues for further analysis.

ApoE^−/−^ mice on a C57BL6/J background (The Jackson Laboratory) fed with HFHCD (Research diets, catalog no. D12079B) were treated with FT3, LT3, or PLT3, or saline control as described above for 2 months to evaluate the effect of different T3 formulations on vascular inflammation and atherosclerosis.

13-week-old male lipodystrophic mice caused by adipocyte-specific ablation of MDM2^66^ were used to test whether the metabolic benefits of PLT3 are dependent on adipose tissues. Mice were randomly divided into two groups for administration with saline or PLT3 at a T3 dose of 50 μg/kg every other day for 32 days. After treatment, body weight, serum levels of insulin and TC, and hepatic TG were measured. In another set of experiments, Ucp1 knockout mice (Ucp1 KO) (The Jackson Laboratory) on C57BL6/J background were fed with HFD for 2 months, followed by treatment with PLT3 at a T3 dose of 50 μg/kg or saline control every other day for 24 days. Body weight, body composition, energy expenditure, and OCR in iWAT were measured as described above.

### Optimization of PTP ratios in liposomes for targeting adipocytes in vitro

Stromal vascular fractions (SVFs) were isolated from iWAT of 6-week-old male C57BL/6 mice. Briefly, subcutaneous fat pad was digested with type I collagenase (Invitrogen, catalog no. 17100017) at a concentration of 0.1% (w/v) and incubated at 37 °C. After 1 h, the digestion mixture was passed through a 100‐μm cell strainer (Merck, catalog no. CLS431752) and centrifuged at 800 × *g* for 10 min. The pelleted SVF was collected and were subjected to adipocyte differentiation by sequential treatment with 0.5 mM isobutylmethylxanthine, 2 µg/ml dexamethasone, and 10 μg/ml insulin for 48 h, followed by treatment with 10 μg/ml insulin only for 6 days. Mature adipocytes at day 8 after differentiation were incubated with LCy5 and PLCy5 with 2%, 5%, 10% of PTP at a Cy5 dose of 50 µmol/mL for 24 h, or preincubated with PTP at a final concentration of 2 mmol/ml for 4 h, followed by incubation with 5%PLCy5 for 24 h. Afterwards, the medium was removed and the cells were rinsed twice with PBS, followed by trypsinization and resuspension in PBS. Fluorescent intensity (Excitation wavelength: 650 nm, Emission wavelength: 670 nm) was determined by flow cytometry (Beckman Coulter, model CytoFLEX LX). To determine the effects of different T3-encapsulated liposomes on browning of white adipocytes, FT3, LT3, 2%, 5%, 10% PLT3 at a T3 dose of 100 nM were included in the culture medium throughout the differentiation process. After 8 days, the mRNA level of browning markers including *Ucp1* and *Pcg1α* were quantified by real-time PCR analysis.

### Determination of tissue distribution of different liposomal particles in mice

8-week-old male C57BL/6 N mice were administered with free Cy5 (FCy5), LCy5, and PLCy5 at a Cy5 dose of 1 μmol/kg by intraperitoneal injection. The mice were sacrificed at various time points after injection. iWAT, eWAT, iBAT, liver, spleen, heart, lung, kidney, brain, and soleus were collected and washed three times with Hank’s Buffered Salt Solution. The fluorescence intensity (Ex 650 nm, Em 670 nm) of tissue homogenates was quantified with a plate reader (BMG LABTECH, model 430-101). The ex vivo fluorescence signal was detected by PE IVIS Spectrum (PerkinElmer, model 124262).

For measurement of T3 concentrations in different tissues, 8-week-old male C57BL/6 N mice were injected with saline, FT3, LT3, or PLT3 at a dose of 2 μg per mouse. The mice were sacrificed at 8 h after injection. iWAT, eWAT, iBAT, liver, spleen, heart, lung, kidney, brain, and soleus were collected and homogenized. T3 concentrations in tissue homogenates were quantified with a commercial ELISA kit (Invitrogen, USA). Results were presented as fold change of T3 level relative to endogenous T3 level in each tissue of saline-treated mice.

### Measurement of heart rate and echocardiography

Heart rate was measured using a non-invasive computerized tail-cuff system (VisiTech Systems, model BP-2000) as previously described^67^. Briefly, mice were placed on a platform equipped with restrainers. Each tail was placed into a computer-controlled tail-cuff. Mice were trained for 3 consecutive days in the prewarmed (37 °C) tail-cuff device to accustom them to the procedure, followed by heart rate measurement. In vivo cardiac morphology and function of mice were assessed using a high-frequency, high-resolution echocardiography system consisting of an ultrasound machine (Visual Sonics, model Vevo 2100) equipped with a transducer (Visual Sonics, model MS250)^68^. Mice were lightly anesthetized with 1% isoflurane and fixed on a heated platform, monitoring the heart rate and temperature. After stabilizing body temperature (37 °C) and heart rate (400 BPM), LV wall thicknesses including LVAW, LVPW, and LVID were analyzed using M-mode imaging from measurements of six separate cycles. LV mass, LV volume, and LVEF were assessed using M-mode.

### Oxygen consumption

Whole-body oxygen consumption (VO_2_) was measured using the comprehensive laboratory animal monitoring system (Columbus Instruments, model Oxymax) as previously described^69^. Briefly, mice were housed individually in CLAMS cages and acclimatized for 24 h. VO_2_ was recorded every 10 min for 72 h at 23 °C.

Ex vivo oxygen consumptions in various tissues were analyzed using a Seahorse Extracellular Flux Analyzer (Agilent Technologies, model XFe24) as previously described^70^. Briefly, tissues were cut into pieces of ~2 mm^3^, weighed, and equilibrated in Dulbecco’s modified eagle medium without NaHCO_3_ at 37 °C for 1 h. The basal oxygen consumption rates (OCRs) in different tissues were recorded and normalized with tissue weight.

### Histological and immunochemistry analysis

For H& E staining, tissues of mice were fixed in 10% buffered formaldehyde, dehydrated by increasing concentrations of alcohol, mounted in xylene, and then immersed in paraffin. The paraffin blocks were cut into 5 μm sections, deparaffinized in xylene, rehydrated. Slides were rinsed in distilled water and then stained with hematoxylin and eosin (H&E). Adipocytes sizes were quantified using ImageJ 1.33 software with the adiposoft plugin. For immunohistochemistry staining, the slides of WAT were incubated overnight with a 1:500 dilution of rabbit monoclonal anti-UCP1 antibody (Abcam, catalog no. ab234430, clone no. EPR23004-34) or a 1:100 dilution of rabbit monoclonal anti-F4/80 antibody (Abcam, catalog no. ab111101, clone no. SP115), washed, and incubated with HRP-conjugated anti-rabbit IgG antibody (CST, catalog no. 7074) at room temperature for 1 h. After staining with 3,3’-Diaminobenzidine (Sigma-Aldrich, catalog no. D4293), images were taken with an optical microscope (Nikon, model Eclipse Ni-U).

### RNA extraction and quantitative real-time PCR

The total RNA was extracted from tissues using TRIzol reagent (Invitrogen, catalog no. 15596026). cDNA was synthesized from RNA (500 ng) by reverse transcription with random primers using a PrimeScript RT reagent kit (Takara, catalog no. RR037A). Real-time PCR mixtures were prepared, including 25 ng cDNA, 5ul SYBR Premix Ex Taq (Takara, catalog no. RR420D), and 250 nM each primer (Invitrogen, listed in Supplementary Data 1). All reactions were performed in triplicate on the Applied Biosystems Prism 7000 sequence detection system. The relative abundance of each gene was normalized against the mRNA level of the ribosomal protein S18 (Rps18).

### Western blot

Total proteins in iWAT and eWAT lysates were extracted using RIPA buffer (Pierce, catalog no. VG297176) and quantified using an enhanced BCA protein assay kit (Thermo, catalog no. 23221). Equal amount of proteins were separated by 12% SDS-PAGE, transferred onto polyvinylidene difluoride membranes (Millipore, catalog no. IPVH00010), sequentially incubated with a 1:1000 dilution of rabbit monoclonal anti-UCP1 antibody (Abcam, catalog no. ab234430, clone no. EPR23004-34) or a 1:1000 dilution of rabbit monoclonal anti-throsine hydroxylase (THA) antibody (Cell signaling technology, catalog no. 58844) at 4 °C overnight. After washing several times, membranes were incubated with HRP-conjugated anti-rabbit IgG antibody (CST, catalog no. 7074) at room temperature for 1 h, followed by addition of ECL detection solution. The protein bands were visualized by a ChemiDoc Touch Imaging System (Bio-Rad, model 1708370). The representative images were processed from uncropped blots (Supplementary Fig. 7). and quantitatively analyzed using ImageJ 1.33 software. β-tubulin expression was used as a loading control.

### Atherosclerosis lesion analysis

The area of atherosclerotic lesions was assessed as previously described^71^. Briefly, the aorta and the upper portion of the heart were excised and immediately fixed in 10% formalin. For en face analysis of lesions, the aortic arch was dissected out, opened longitudinally, pinned on a black wax pan, and stained with Oil red O overnight. The images of the aortic arch were captured using an optical microscope (Nikon, model Eclipse Ni-U) and analyzed with ImageJ 1.33 software. For analysis of plaque lesion in aortic sinus, the heart was removed and embedded in OCT compound. Serial 10 μm-thick cryosections from the middle portion of the ventricle to the aortic arch were collected. Cryosections of aortic sinus were stained with Oil Red O for 15 min. The lipid-containing area on each section was determined in a blinded fashion using an optical microscope (Nikon, model Eclipse Ni-U). For immunofluorescence staining, aortic root sections were incubated overnight with a mixture of rabbit monoclonal anti-α-smooth muscle actin antibody (α-SMA, Abcam, catalog no. ab124964, clone no. EPR5368, 1:250 dilution) and rat monoclonal anti-monocyte/macrophage-2 antibody (MOMA-2, Abcam, catalog no. ab33451, clone no. MOMA-2, 1:250 dilution), washed, and incubated with a mixture of alexa fluor 488-conjugated anti-rabbit IgG antibody (Invitrogen, catalog no. A11034 for α-SMA) and alexa fluor 594-conjugated anti-rat IgG antibody (Invitrogen, catalog no. A11007 for MOMA-2) at room temperature for 1 h. The nucleus was stained with DAPI (Sigma-Aldrich, catalog no. D9542). Images were taken with a fluorescence microscope (Nikon, model Eclipse Ts2).

### Micro-CT imaging

Femurs dissected from mice were fixed in 3.5% formaldehyde for 24 h before being scanned and analyzed with a micro-CT system (Bruker, model Skyscan 1272). Two phantoms (0.25 g/cm^3^ and 0.75 g/cm^3^ stoichiometric hydroxyapatite in epoxy resin, 4 mm diameter) were scanned to calculate the trabecular BMD^72^. Bone volume (BV) fraction was calculated by triangulating the surface of the trabecular bone and normalized with the total volume (TV) of the examined femur. Trabecular number (Tb. N) was defined as the inverse of the mean distance between the mid-axes of the structure. Trabecular thickness (Tb. Th) was obtained by filling the maximum size of spheres in the structure.

### Biomechanical analysis of femur

The mouse femurs were subjected to a three-point bending test with MTS 858 Mini Bionix II Testing System (MTS, USA). The femurs were immobilized on a fixed support with two loading points with a 9.5-mm interval distance. The upper loading point was located at the midpoint between the two lower loading points. Afterwards, the load was applied at a constant speed until bone fracture occurred. The inner and outer width and height of the femur at the point of fracture were measured with a vernier caliper. The ultimate force was determined from the force-displacement curve. Elastic modulus was calculated according to the formula^73^:

2 $$E={{KL}}^{3}/48I$$

where *K* is the stiffness, *L* is the distance between supporting points, and *I* is the moment of inertia of the cross-section in relation to the horizontal axis.

### Biochemical and immunological analysis for serum parameters

Serum parameters including total TG, TC, HDL-C, LDL-C, T3, norepinephrine, cAMP, TNF-α, IL-6, MCP-1, TSH, insulin, adiponectin, ALT, and ALT were measured with commercial kits (Stanbio, catalog no. 2100 for TG; Stanbio, catalog no. 1010 for TC; Stanbio, catalog no. 0950 for HDL-C; FineTest, catalog no. EU0403 for T3; Abnova, catalog no. KA1891 for norepinephrine; Novusbio, catalog no. KA0886 for cAMP; R& D systems, catalog no. DY410 for TNF-α; R& D systems, catalog no. DY406 for IL-6; R& D systems, catalog no. DY479 for MCP-1; LSBio, catalog no. LS-F22889 for TSH; Immunodiagnostics, catalog no. 32270 for insulin; Immunodiagnostics, catalog no. 32010 for adiponectin; Stanbio, catalog no. 2930 for ALT; Stanbio, catalog no. 2920 for AST). Liver total lipids were extracted with acetone and chloroform/methanol (2/1, v/v) as described^74^, and total TG, TC, and FFA levels in liver were quantified with commercial kits (Roche, catalog no. 11383175001 for FFA) and normalized by liver weight (g).

### Statistical analysis

Unpaired student’s *t* test was used for comparison of two experimental conditions with normal distribution. One-way analysis of variance (ANOVA) followed by least significant difference (LSD) test was applied for comparisons of more than two groups. Two-way ANOVA followed by Tukey’s test was used for groups with different genotypes and treatments as factors. Analysis of covariance (ANCOVA) was used for comparisons of whole-body oxygen consumption (VO_2_) of mice among multiple groups using body weight as a covariate as previously described^75^. One-way ANOVA and ANCOVA were performed with Statistical Package for Social Sciences version 14.0 (SPSS, Chicago. IL). Unpaired student’s *t* test and two-way ANOVA were performed with GraphPad Prism software version 8.2.1 (GraphPad Software). Data were expressed as mean ± SEM. All *p* values are two-sided and *p* < 0.05 were considered statistically significant.

### Reporting summary

Further information on research design is available in the Nature Portfolio Reporting Summary linked to this article.

## Supplementary information

Supplementary Information

Description of Additional Supplementary Files

Supplementary Data 1

Reporting Summary

## Source data

Source Data

## Data Availability

All data supporting the findings described in this manuscript are available in the article and in the Supplementary Information and from the corresponding author upon request. Source data are provided with this paper.
