# Author Correction: Adipose-targeted triiodothyronine therapy counteracts obesity-related metabolic complications and atherosclerosis with negligible side effects

**DOI:** 10.1038/s41467-026-71985-w

**Published:** 2026-04-13

**Authors:** Kang Chen, Lai Yee Cheong, Yuan Gao, Yaming Zhang, Tianshi Feng, Qin Wang, Leigang Jin, Eric Honoré, Karen S. L. Lam, Weiping Wang, Xiaoyan Hui, Aimin Xu

**Affiliations:** 1 State Key Laboratory of Pharmaceutical Biotechnology, The University of Hong Kong, Pokfulam, Hong Kong China; 2 Department of Medicine, Li Ka Shing Faculty of Medicine, The University of Hong Kong, Hong Kong, China; 3 Dr Li Dak-Sum Research Centre, The University of Hong Kong-Karolinska Institutet Collaboration in Regenerative Medicine, The University of Hong Kong, Pokfulam, Hong Kong China; 4 Department of Pharmacology & Pharmacy, Li Ka Shing Faculty of Medicine, The University of Hong Kong, Pokfulam, Hong Kong China; 5 Université Côte d’Azur, Centre National de la Recherche Scientifique, Institut National de la Santé et de la Recherche Médicale, Institut de Pharmacologie Moléculaire et Cellulaire, Labex ICST, Valbonne, France

Correction to: *Nature Communications* 10.1038/s41467-022-35470-4, published online 20 December 2022

In the version of this article initially published, due to a figure assembly error, in Fig. 6c, upper panel, the representative H&E staining image for LT3 was inadvertently chosen from the Saline group. This correction does not affect the study’s results or conclusions. The figure is now amended in the HTML and PDF versions of the article.

