# Supplementary material for: Adipose-targeted triiodothyronine therapy counteracts obesity-related metabolic complications and atherosclerosis with negligible side effects
Source: Nat Commun. 2022 Dec 20;13:7838. doi: 10.1038/s41467-022-35470-4 (PMC9767940; doi:10.1038/s41467-022-35470-4)
Supplement: Supplementary file 1 — Supplementary Information [file 41467_2022_35470_MOESM1_ESM.pdf]

SUPPLEMENTARY INFORMATION

Adipose-Targeted Triiodothyronine Therapy Counteracts Obesity-Related Metabolic Complications and Atherosclerosis with Negligible Side Effects

Supplementary Figure 1

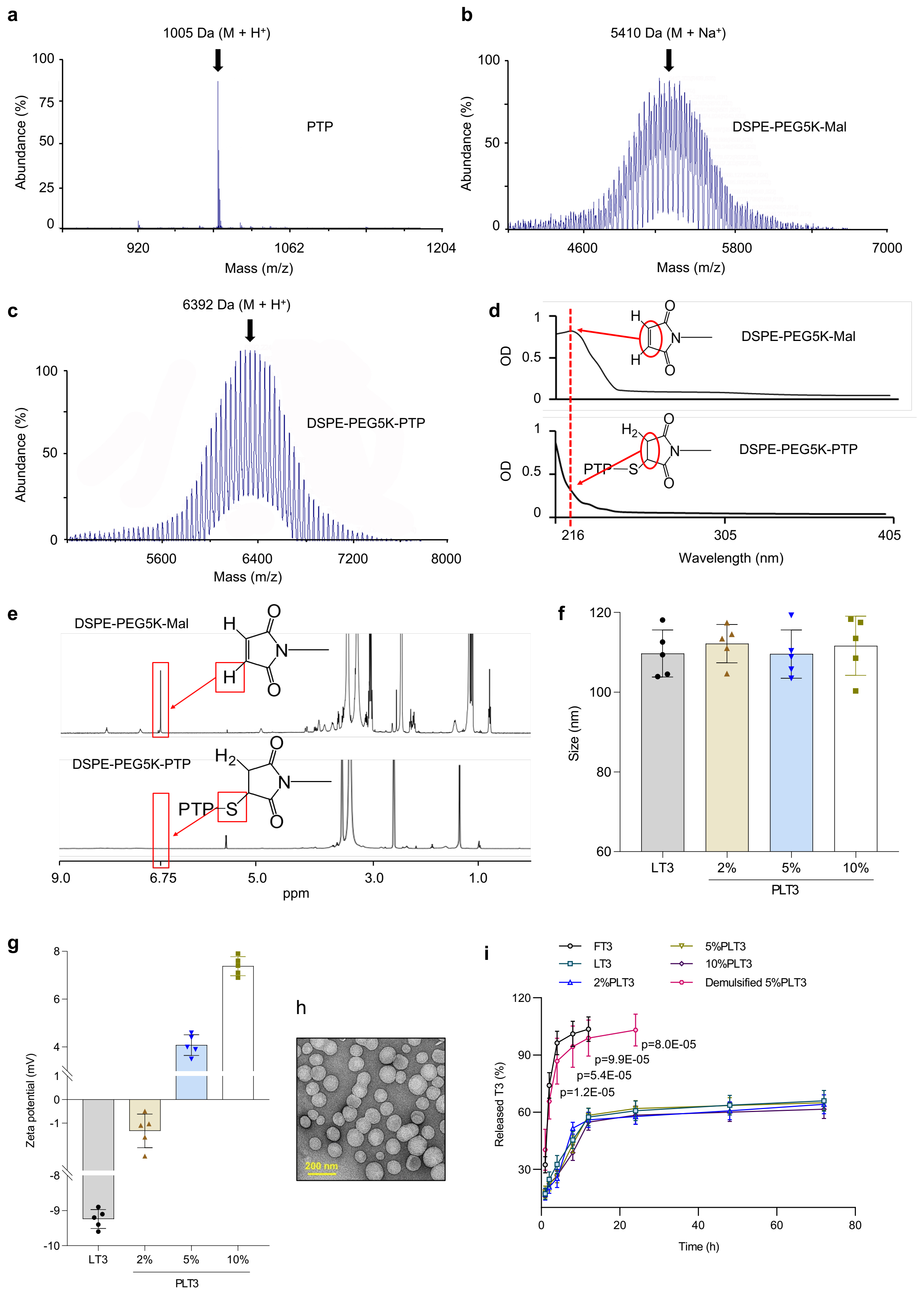

Supplementary Figure 1. **Characterization of liposomal nanoparticles synthesized in this study.** (**a-c**) MALDI-ToF spectrums of PTP (**a**), DSPE-PEG5K-Mal (**b**), and DSPE-PEG5K-PTP (**c**). (**d**) Ultraviolet-visible (UV) spectrum and (**e**) proton nuclear magnetic resonance (^{1}H-NMR) spectrum of DSPE-PEG5K-Mal and DSPE-PEG5K-PTP. (**f**) Size and (**g**) zeta potential of liposomes measured with a dynamic scattering laser. (**h**) Visualization of PLT3 with 5 mol % PTP of total lipids (5%PLT3) by a transmission electron microscope. (**i**) The in vitro release profile of T3 from free T3 (FT3), LT3, 2%PLT3, 5%PLT3, 10%PLT3, and demulsified 5%PLT3. Unpaired student's t test was used for comparison of release profiles of 5%PLT3 and demulsified 5%PLT3. All data are expressed as mean ± SEM. N = 5 independent replicates from one experiment. P values were two-sided. Source data are available as a Source Data file.

Supplementary Figure 2

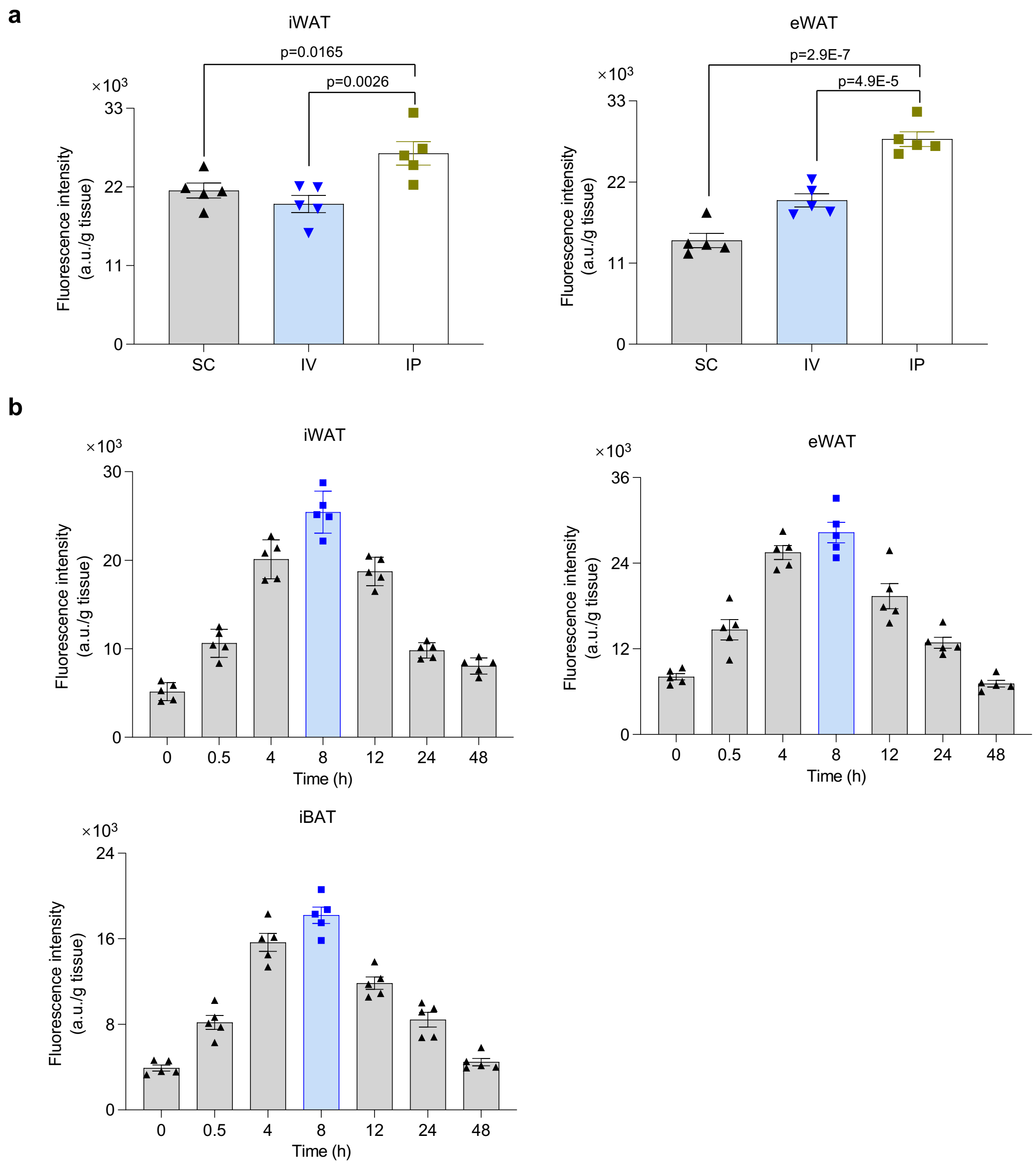

Supplementary Figure 2. Uptake of liposomes by WAT via different administration routes and time course of fluorescence intensity in WAT after injection of liposomes. (**a**) 8-week-old male C57BL/6N mice were administrated with PLCy5 via subcutaneous (SC), intravenous (IV), or intraperitoneal (IP) injection. The relative fluorescence intensity in iWAT and eWAT was determined 8 hours after injection. (**b**) 8-week-old male C57BL/6N mice were IP injected with PLCy5. The relative fluorescence intensity in iWAT, eWAT, and iBAT was determined at various time points after the injection. All data are expressed as mean ± SEM. N = 5 biologically independent replicates from one experiment. One-way ANOVA followed by Least Significant Difference (LSD) test was applied for comparisons among multiple groups. All the p values were two-sided. Source data are available as a Source Data file.

Supplementary Figure 3

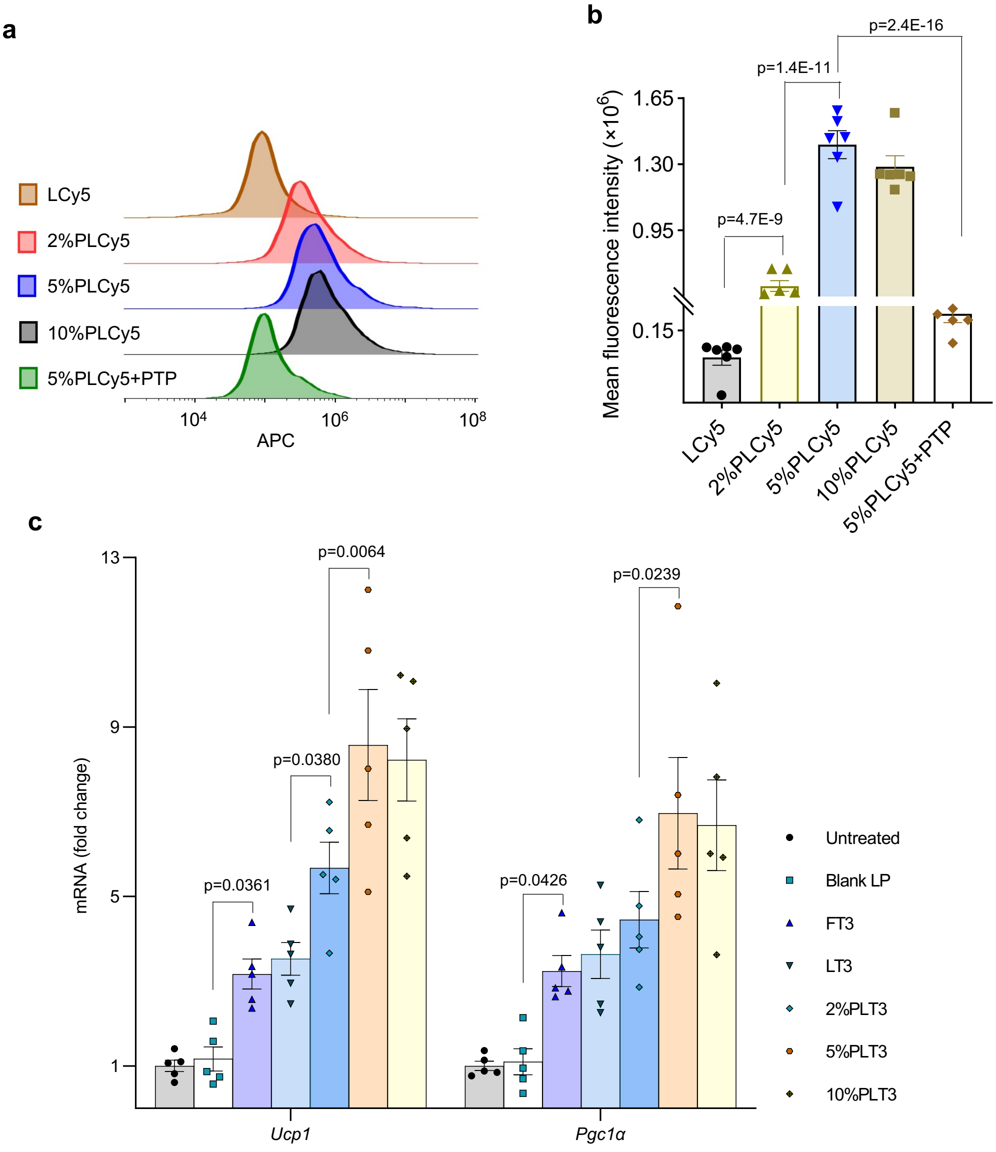

Supplementary Figure 3. Optimization of PTP ratio in liposomes in mouse adipocytes. Mouse adipocytes were differentiated from stromal vascular fraction (SVF) cells isolated from subcutaneous WAT (iWAT) of 6-week-old C57BL/6 mice. (**a**,**b**) Cell uptake of liposomes with different PTP ratios by adipocytes. Mouse adipocytes were incubated with LCy5 and PLCy5 with 2%, 5%, 10% of PTP at a Cy5 dose of 50 µmol/mL for 24 hours, or preincubated with PTP at a concentration of 2 mmol/ml for 4 hours, followed by incubation with 5%PLCy5 for 24 hours. Flow cytometry analysis was applied to determine cellular uptake. Mean fluorescence intensities were generated by a FlowJo software. (**c**) The effect of liposomes without or with different PTP ratios on browning of white adipocytes. SVF cells were differentiated to beige adipocytes in the presence of FT3, LT3, 2%, 5%, 10% PLT3 at a T3 dose of 100 nM, with saline and PTP-decorated blank liposomes (Blank PL) as control. Gene expressions of *Ucp1* and *Pgc1α* were measured with real-time PCR analysis. N = 5 from one experiment. In **b**, n = 6 biologically independent replicates from one experiment. In **c**, n = 5 biologically independent replicates from one experiment. Data are expressed as mean ± SEM. One-way ANOVA followed by LSD test was applied for comparisons among multiple groups. All the p values were two-sided. Source data are available as a Source Data file.

Supplementary Figure 4

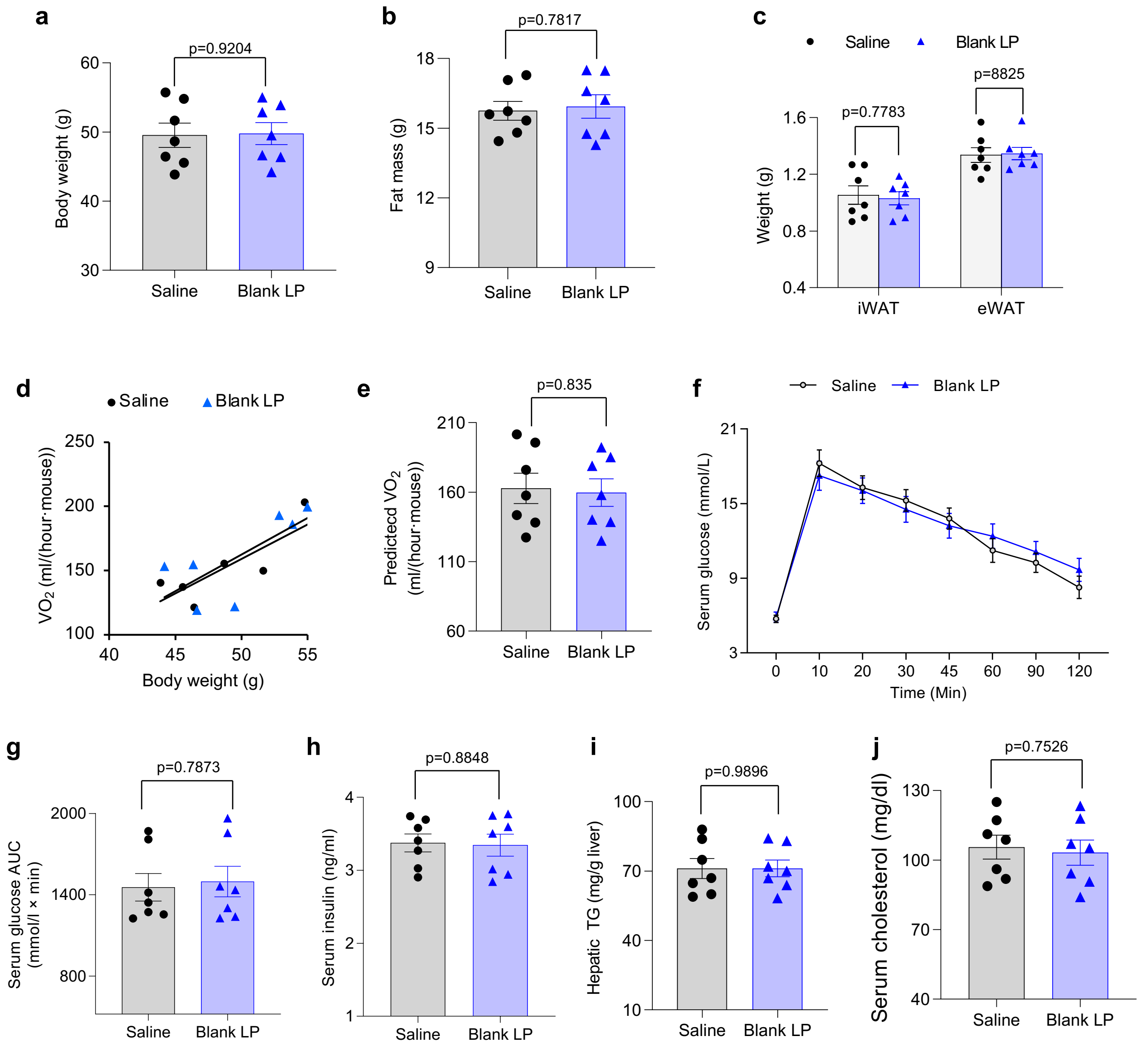

Supplementary Figure 4. Blank liposomal particles have no significant effects on body weight, energy expenditure, glucose and lipid profiles, insulin and fatty liver in C57BL/6N mice. 8-week-old male C57BL/6N mice mice were fed with high-fat diet (HFD) for 8 weeks followed by intraperitneal injection with saline or 5% PTP-decorated blank liposomes (Blank LP) at a dose of 12.2 mg/kg every two days for 32 days. (**a**) Comparison of body weight between two groups. (**b**) Fat mass of mice measured by a Body Composition Analyzer. (**c**) Wet weight of iWAT and eWAT. (**d**,**e**) Whole-body VO_2 of mice after treatment was measured with the Comprehensive Lab Animal Monitoring System. (**d**) Regression plots of whole-body oxygen consumption rate (VO_2) against body weight. (**e**) ANCOVA-predicted whole-body VO_2 at the mean body weight (49.53 g) of saline-treated mice. (**f**,**g**) Glucose excursion curve (**f**) and area under the curve (**g**) for ipGTT. (**h**) Fasting serum level of insulin. (**i**) The content of triglyceride in the liver. (**j**) Fasting serum level of total cholesterol. All data are expressed as mean ± SEM. N = 7 biologically independent replicates from one experiment. Unpaired **s**tudent's t test was applied for comparison between two groups. All the p values were two-sided. Source data are available as a Source.

Supplementary Figure 5

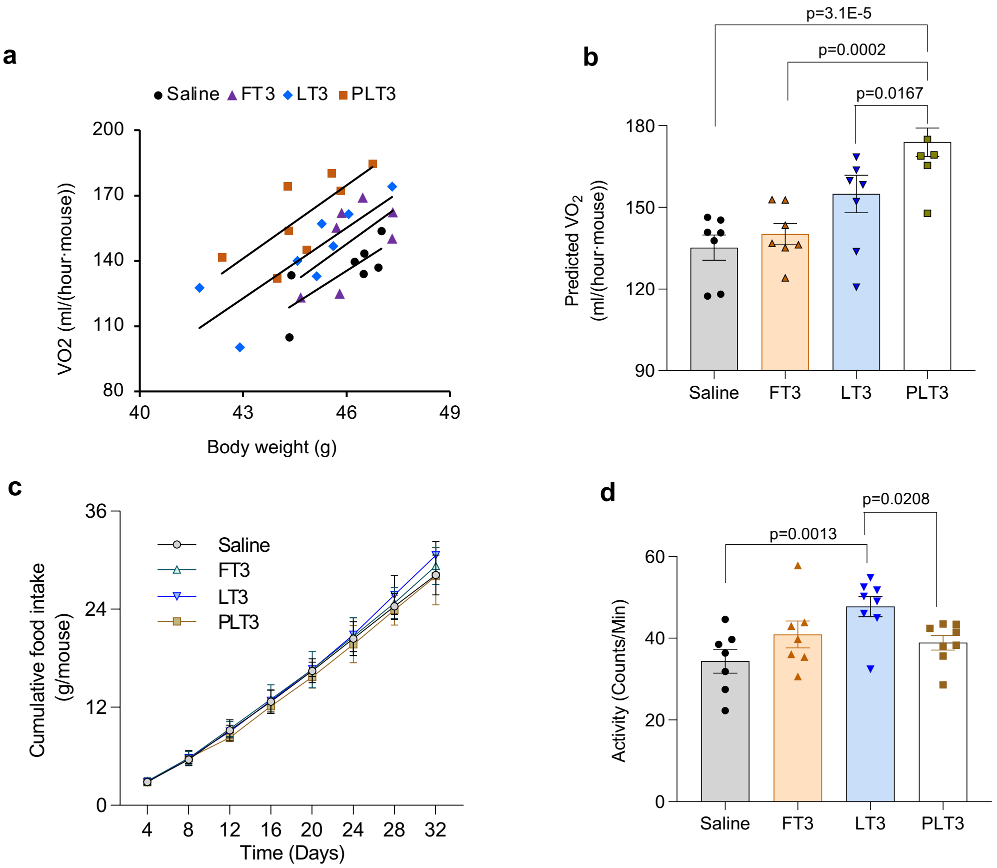

Supplementary Figure 5. Effects of FT3, LT3 and PLT3 on whole-body oxygen consumption (VO_2), cumulative food intake, and locomotor activity in mice. HFD-induced male obese mice were intraperitoneally administered with different forms of T3 or saline for 32 days as in Figure 2. (**a-b**) Whole-body VO_2 of mice at 14 days after treatment was measured with the Comprehensive Lab Animal Monitoring System. (**a**) Regression plots of whole-body oxygen consumption rate (VO_2) against body weight. (**b**) ANCOVA-predicted whole-body VO_2 at the mean body weight (45.98 g) of saline-treated mice. (**c**) Cumulative food intake of mice. (**d**) Locomotory activity (XAMB) of mice was measured. All data are expressed as mean ± SEM. N = 7 (Saline and FT3) or 8 (LT3 and PLT3) biologically independent replicates from one experiment. One-way ANOVA followed by LSD test was applied for comparisons among multiple groups. All the p values were two-sided. Source data are available as a Source Data file.

Supplementary Figure 6

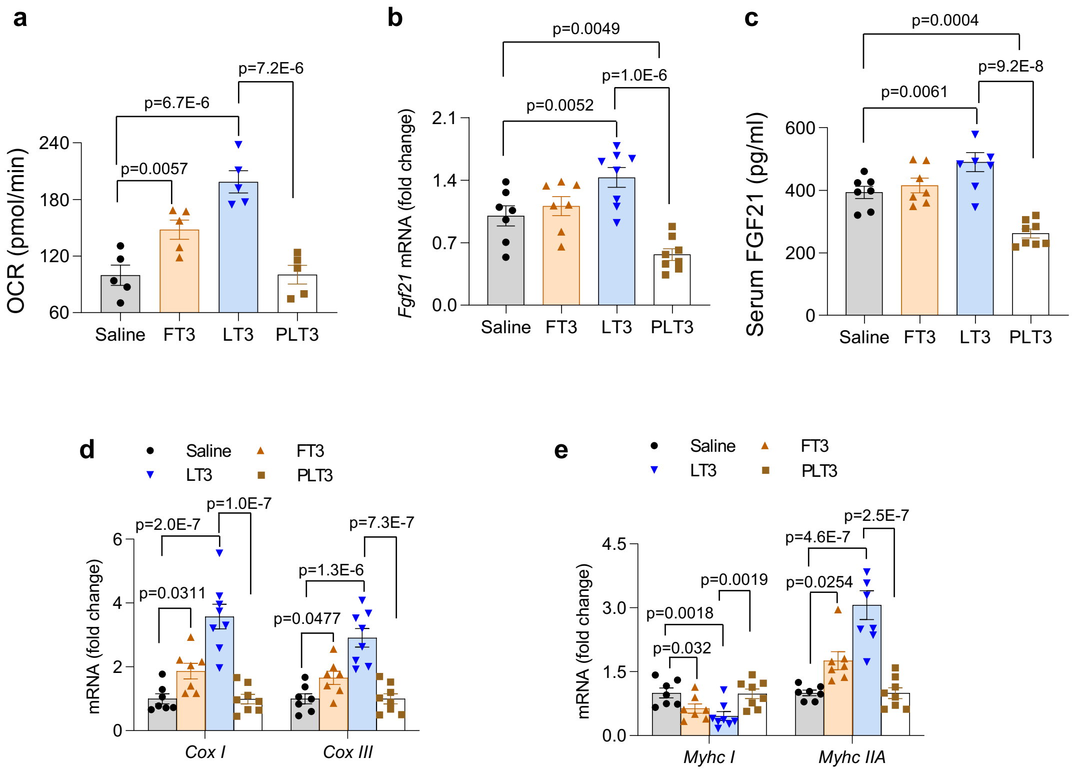

Supplementary Figure 6. Effect of different T3 formulations on hepatic oxygen consumption, FGF21 production, and gene expression in skeletal muscle. HFD-fed male C57BL/6N mice were treated with different forms of T3 or saline for 32 days as in Figure 2. (**a**) Oxygen consumption rate (OCR) in liver measured by a Seahorse bioanalyzer. (**b**) The mRNA abundance of *Fgf21* in liver quantified by real-time PCR. (**c**) Serum FGF21 levels measured with ELISA. (**d,e**) The mRNA level of mitochondrial DNA (mtDNA)-encoded *cytochrome c oxidase subunit I (Cox I)* and *cytochrome c oxidase subunit 1 (Cox III),* and *myosin heavy chain I (Myhc I)*, *myosin heavy chain IIA (Myhc IIA)* in soleus muscle. All data are expressed as mean ± SEM. N = 7 (Saline and FT3) or 8 (LT3 and PLT3) biologically independent replicates from one experiment. One-way ANOVA followed by LSD test was applied for comparisons among multiple groups. All the p values were two-sided. Source data are available as a Source Data file.

Supplementary Figure 7

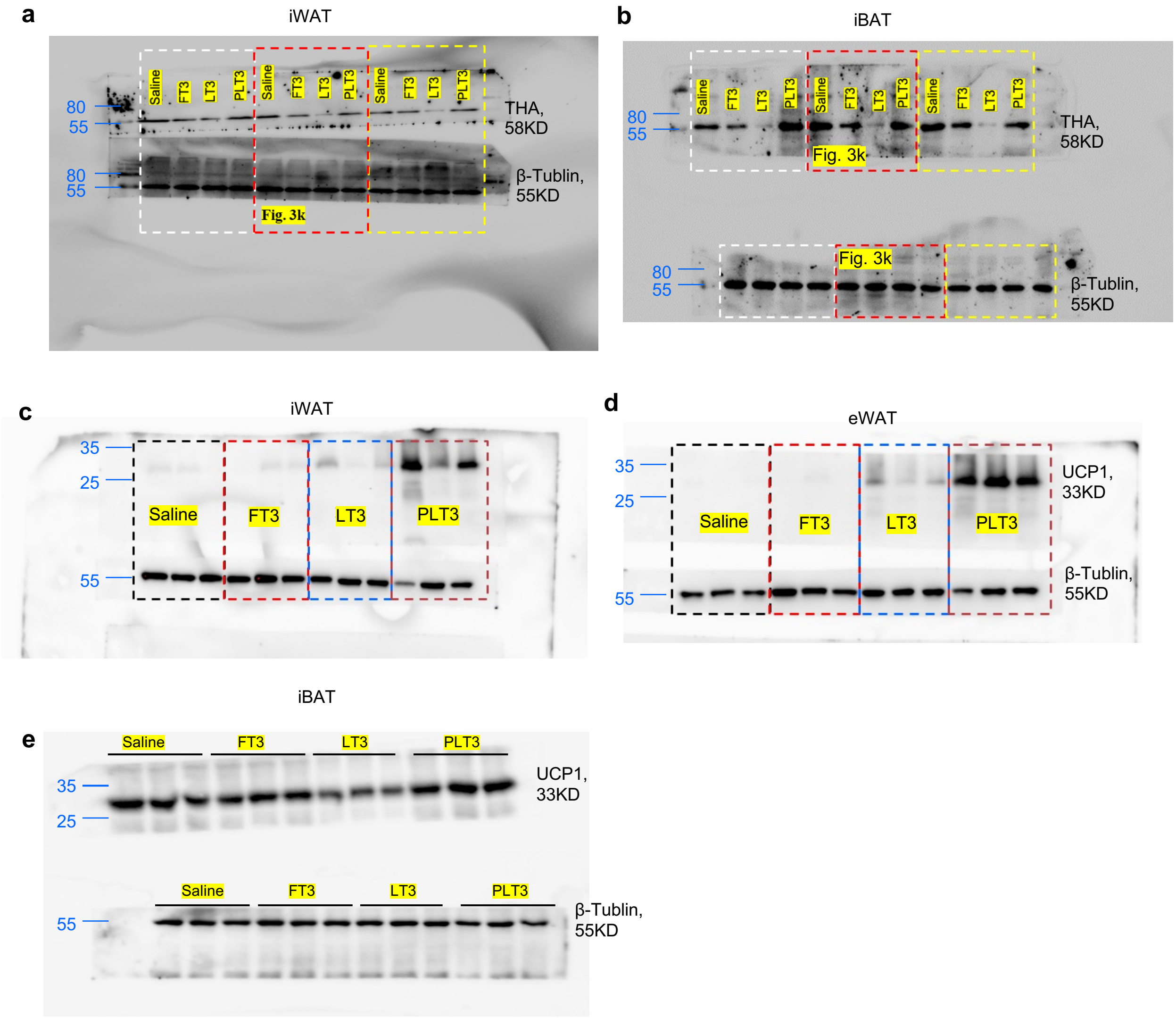

Supplementary Figure 7. Scans of uncropped immunoblots. (**a,b**) The scan of uncropped immunoblots of Figure 3k. (**c,e**) The scan of original immunoblots of Figure 4d.

Supplementary Figure 8

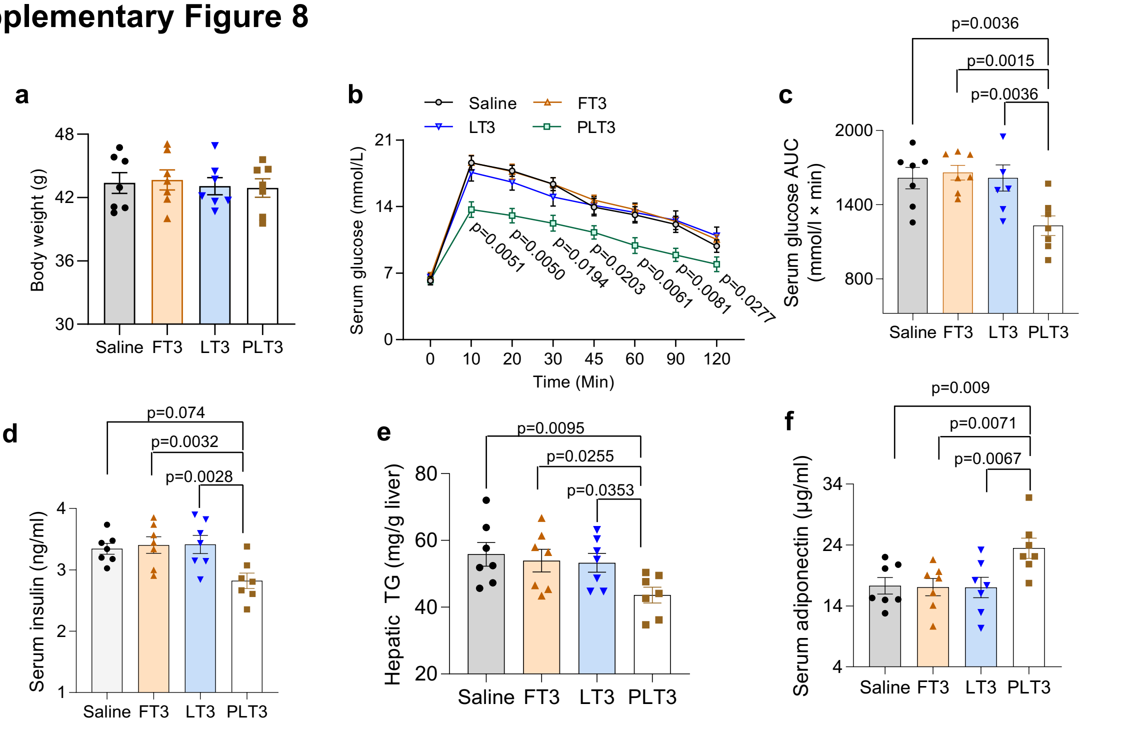

Supplementary Figure 8. Metabolic changes in obese mice treated with different formulations of T3 for one week. HFD-fed male C57BL/6N mice were treated with different forms of T3 or saline for 7 days as in Figure 2. (**a**) Body weight of mice. (**b**) Glucose excursion curve for intraperitoneal glucose tolerance test (ipGTT). Data are expressed as mean ± SEM. (**c**) Area under the curve for (**b**). (**d**) Fasting serum level of insulin. (**e**) The triglyceride (TG) contents in the liver. (**f**) Serum level of adiponectin. All data are expressed as mean ± SEM. N = 7 biologically independent replicates from one experiment. One-way ANOVA followed by LSD test was applied for comparisons among multiple groups. All the p values were two-sided. Source data are available as a Source Data file.

Supplementary Figure 9

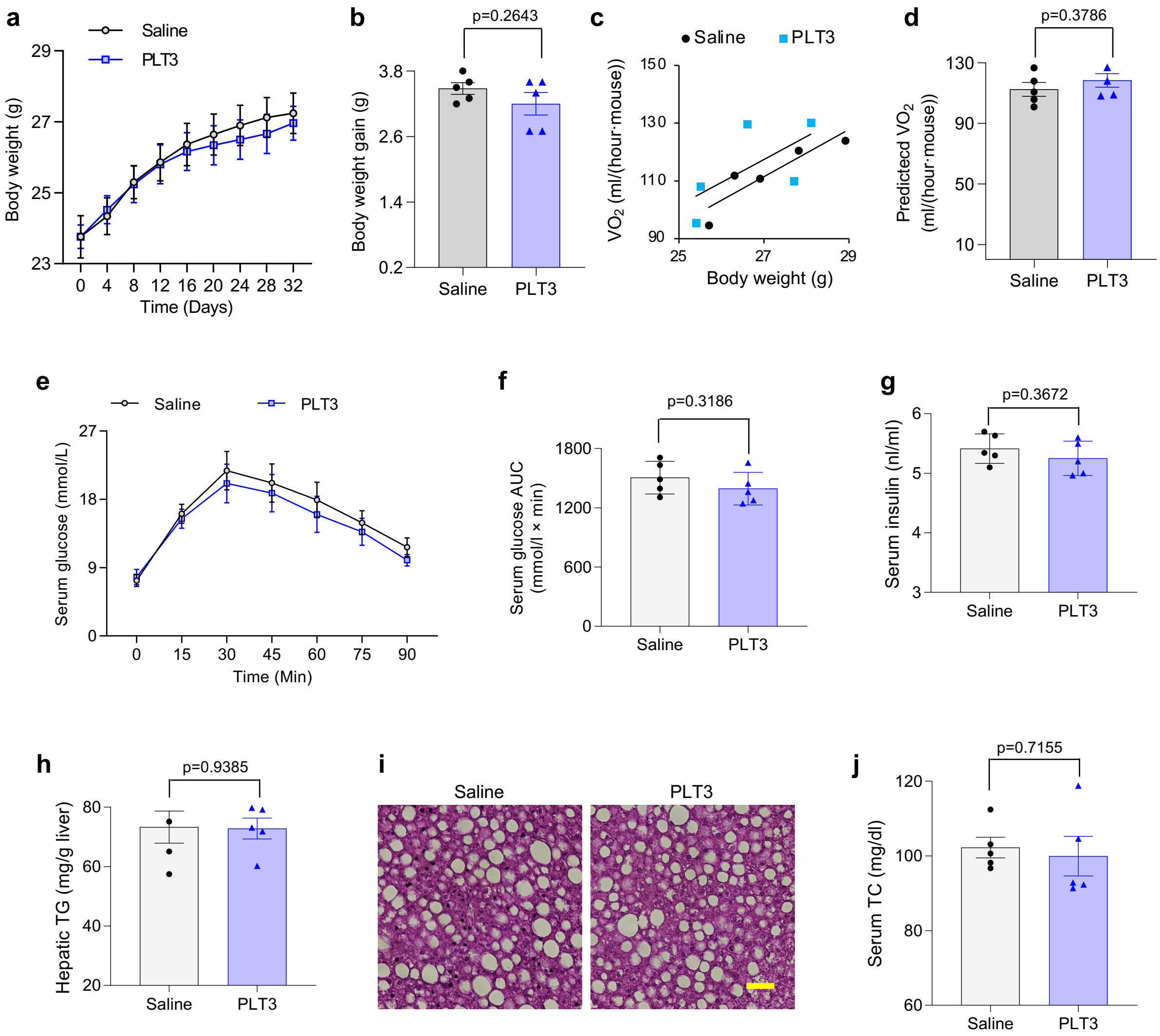

Supplementary Figure 9. The metabolic benefits of PLT3 are abrogated in lipodystrophic mice without adipose tissues. 13-week-old male Adipo-MDM2-KO mice were intraperitoneally injected with PLT3 or saline every two days for 32 days. (**a**) Dynamic changes in body weight during the treatment. (**b**) Comparison of body weight gain between different groups from the 0th day to the 32th day. (**c**,**d**) Whole-body VO_2 of mice measured at 28 days after treatment. (**c**) Regression plots of whole-body VO_2 against body weight. (**d**) ANCOVA-predicted whole-body VO_2 at the mean body weight (27.12 g) of saline-treated mice. N = 5 from one experiment. (**e**) Glucose excursion curve and (**f**) area under the curve for ipGTT. (**g**) Serum insulin level of mice. (**h**) The triglyceride content in the liver. (**i**) H&E staining of liver sections. Graphs are from one experiment. (**j**) Serum level of total cholesterol. All data are expressed as mean ± SEM. N = 5 biologically independent replicates from one experiment. Unpaired student's t test was applied for comparison between two groups. All the p values were two-sided. Source data are available as a Source Data file.

Supplementary Figure 10

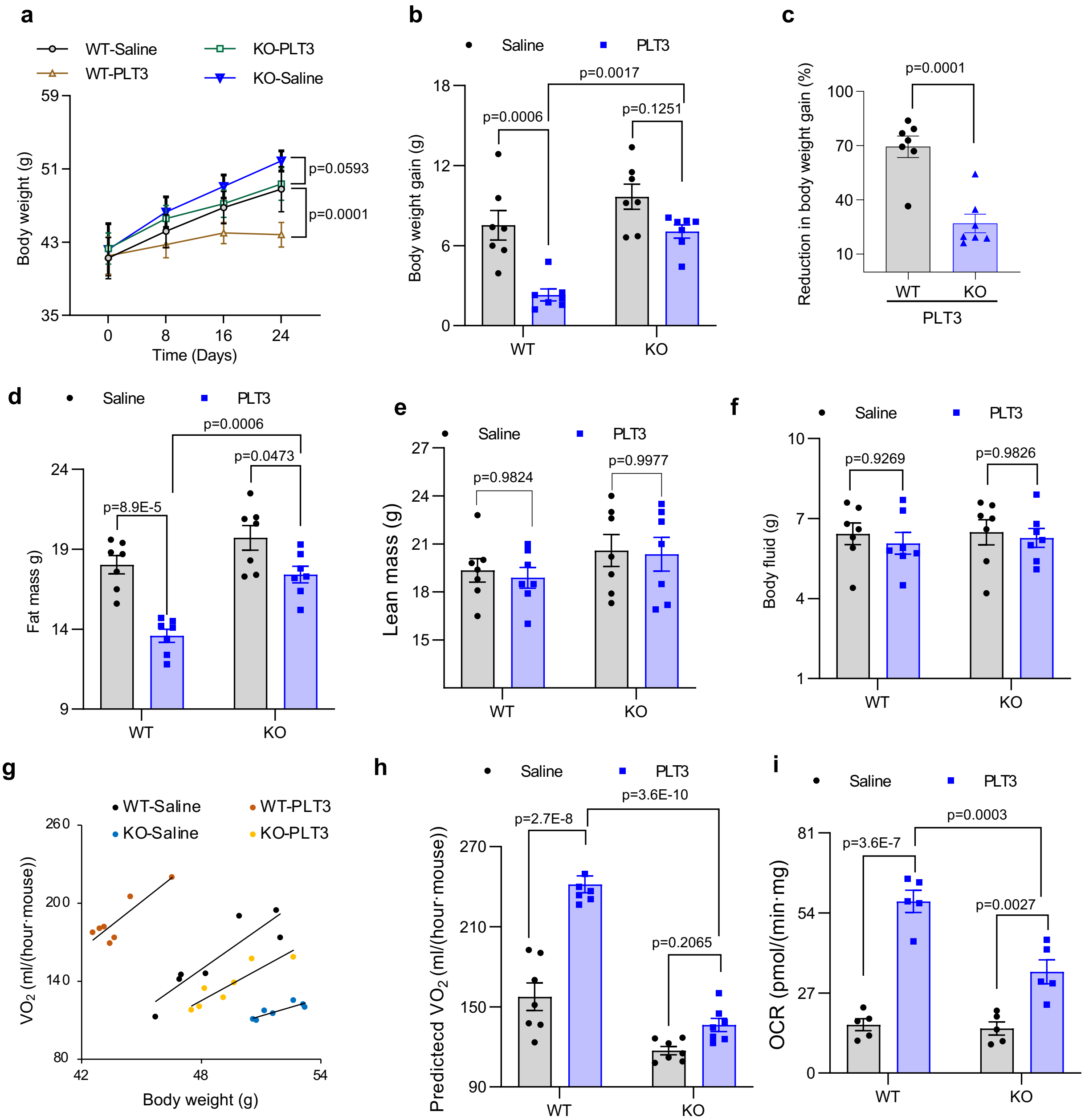

Supplementary Figure 10. Comparison of the effect of PLT3 on body weight, fat mass, energy expenditure, and oxygen consumption in adipose tissue between UCP1 KO mice and WT littermates. 8-week-old male wild-type (WT) and UCP1 KO mice were fed with HFD for 2 months and then intraperitoneally injected with PLT3 or saline every two days for 24 days. (**a**) Dynamic changes in body weight during the treatment. (**b**) Comparison of body weight gain between the two groups during the 24-day treatment period. (**c**) Percentage reduction in body weight gain by PLT3, compared to saline-treated group. (**d-f**) Fat mass (**d**), lean mass (**e**), and body fluid (**f**) were measured with a Body Composition Analyzer. (**g**,**h**) Whole-body VO_2 of mice were measured with the Comprehensive Lab Animal Monitoring System (CLAMS). (**g**) Regression plots of whole-body VO_2 against total body weight. (**h**) ANCOVA-predicted whole-body VO_2 at a common body weight of 48.78 g and 51.87 g for WT and UCP1 KO mice, respectively. (**i**) Oxygen consumption rate (OCR) in iWAT was measured by a Seahorse bioanalyzer. In **a**-**h**, n = 7 biologically independent replicates from one experiment. In **i**, n = 5 biologically independent replicates from one experiment. Data are expressed as mean ± SEM. In **c**, unpaired student's t test was used for comparison between two groups. In **a**, **b**, **e-f**, **h**, and **i**, two-way ANOVA followed by Tukey's test for comparisons among multiple groups. All the p values were two-sided. Source data are available as a Source Data file.

Supplementary Figure 11

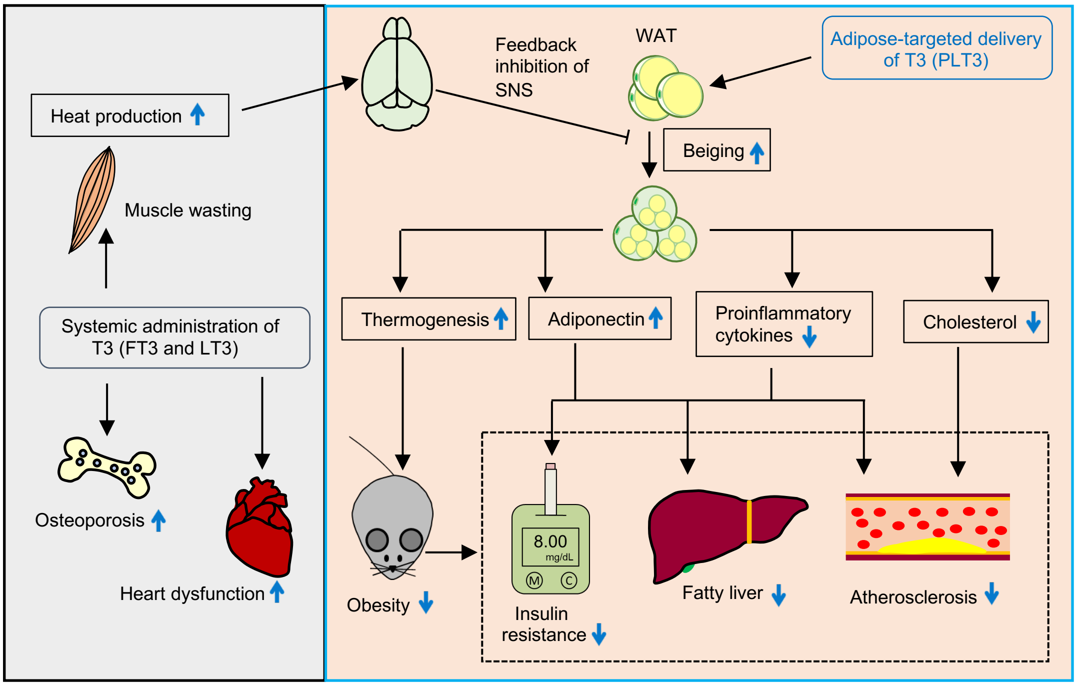

Supplementary Figure 11. A schematic diagram illustrating how adipose-targeted delivery of T3 uncouples its beneficial effects from multiple deleterious effects on non-adipose organs. Chronic, systemic administration of T3 does not induce thermogenesis of beige adipocytes in WAT due to the feedback suppression of sympathetic nerve system (SNS) possibly caused by increased heat production in muscle. Additionally, it causes adverse effects on heart, bone, and muscle. In contrast, T3 delivered with PLT3 is preferentially accumulated in adipose tissues to stimulate biogenesis of beige adipocytes by increasing UCP1 expression without affecting adrenergic input to WAT, thus counteracting obesity by enhancing thermogenesis in WAT. Furthermore, PLT3 remodels WAT to a metabolically healthy phenotype with increased adiponectin production, reduced adipose inflammation, and decreased proinflammatory cytokines/adipokines, consequently alleviating obesity-related insulin resistance, fatty liver, hypercholesterolemia, and atherosclerosis. Furthermore, PLT3 bypasses the adverse effects of systemic administration of T3.

Supplementary Table 1. PLT3 reduces hypercholesterolemia independently on reduction of body weight. $ApoE^{-/-}$ mice on HFHCD were IP injected with different forms of T3 or saline for 5 days. Body weight and serum levels of TC, HDL-C, and LDL-C were measured. N = 7 biologically independent replicates from one experiment. All data used one-way ANOVA followed by LSD test for comparisons among multiple groups. Data are expressed as mean ± SEM. Two-sided p values were for comparing each group with PLT3 group. Source data are available as a Source Data file.

	Body weight (g)	p value vs. PLT3
Saline	31.78 ± 0.70	0.6254
FT3	31.19 ± 0.89	0.9844
LT3	31.08 ± 0.87	0.9096
PLT3	31.21 ± 0.80	___

	TC (mg/dl)	p value vs. PLT3
Saline	331.30 ± 10.75	0.0343
FT3	329.48 ± 17.06	0.0416
LT3	337.26 ± 14.31	0.0178
PLT3	286.77 ± 7.87	___

	HDL-C (mg/dl)	p value vs. PLT3
Saline	75.67 ± 2.51	0.5928
FT3	75.31 ± 2.55	0.6556
LT3	74.45 ± 3.32	0.8121
PLT3	73.47 ± 3.04	___

	LDL-C (mg/dl)	p value vs. PLT3
Saline	231.73 ± 6.98	0.0002
FT3	228.62 ± 5.31	0.0003
LT3	234.40 ± 14.47	9.9E-5
PLT3	174.36 ± 6.79	___
