# Supplementary material for: Adipose-targeted triiodothyronine therapy counteracts obesity-related metabolic complications and atherosclerosis with negligible side effects
Source: Nat Commun. 2022 Dec 20;13:7838. doi: 10.1038/s41467-022-35470-4 (PMC9767940; doi:10.1038/s41467-022-35470-4)
Supplement: Supplementary file 2 — Description of Additional Supplementary Files [file 41467_2022_35470_MOESM2_ESM.pdf]

Title: Supplementary Data 1
Description: The sequences of primers used for real-time PCR analysis
