# Supplementary material for: Adipose-targeted triiodothyronine therapy counteracts obesity-related metabolic complications and atherosclerosis with negligible side effects
Source: Nat Commun. 2022 Dec 20;13:7838. doi: 10.1038/s41467-022-35470-4 (PMC9767940; doi:10.1038/s41467-022-35470-4)
Supplement: Supplementary file 4 — Reporting Summary [file 41467_2022_35470_MOESM4_ESM.pdf]

nature portfolio

Corresponding author(s): Aimin Xu; NCOMMS-21-25850B

Last updated by author(s): Nov 21, 2022

Reporting Summary

Nature Portfolio wishes to improve the reproducibility of the work that we publish. This form provides structure for consistency and transparency in reporting. For further information on Nature Portfolio policies, see our Editorial Policies and the Editorial Policy Checklist.

Statistics

For all statistical analyses, confirm that the following items are present in the figure legend, table legend, main text, or Methods section.

n/a	Confirmed	
[ ]	[x]	The exact sample size (*n*) for each experimental group/condition, given as a discrete number and unit of measurement
[ ]	[x]	A statement on whether measurements were taken from distinct samples or whether the same sample was measured repeatedly
[ ]	[x]	The statistical test(s) used AND whether they are one- or two-sided<br>*Only common tests should be described solely by name; describe more complex techniques in the Methods section.*
[ ]	[x]	A description of all covariates tested
[x]	[ ]	A description of any assumptions or corrections, such as tests of normality and adjustment for multiple comparisons
[ ]	[x]	A full description of the statistical parameters including central tendency (e.g. means) or other basic estimates (e.g. regression coefficient) AND variation (e.g. standard deviation) or associated estimates of uncertainty (e.g. confidence intervals)
[ ]	[x]	For null hypothesis testing, the test statistic (e.g. *F*, *t*, *r*) with confidence intervals, effect sizes, degrees of freedom and *P* value noted<br>*Give P values as exact values whenever suitable.*
[x]	[ ]	For Bayesian analysis, information on the choice of priors and Markov chain Monte Carlo settings
[x]	[ ]	For hierarchical and complex designs, identification of the appropriate level for tests and full reporting of outcomes
[x]	[ ]	Estimates of effect sizes (e.g. Cohen's *d*, Pearson's *r*), indicating how they were calculated

Our web collection on statistics for biologists contains articles on many of the points above.

Software and code

Policy information about availability of computer code

Data collection	n/a
Data analysis	ImageJ 1.33; FlowJo V10

For manuscripts utilizing custom algorithms or software that are central to the research but not yet described in published literature, software must be made available to editors and reviewers. We strongly encourage code deposition in a community repository (e.g. GitHub). See the Nature Portfolio guidelines for submitting code & software for further information.

Data

Policy information about availability of data

All manuscripts must include a data availability statement. This statement should provide the following information, where applicable:
- Accession codes, unique identifiers, or web links for publicly available datasets
- A description of any restrictions on data availability
- For clinical datasets or third party data, please ensure that the statement adheres to our policy

The datasets generated and/or analysed in the current study are available from the corresponding author upon reasonable request.

Field-specific reporting

Life sciences study design

All studies must disclose on these points even when the disclosure is negative.

Sample size	The sample sizes for mice were determined based on the mean value and standard deviation of body weight of high fat diet-induced obese mice in our previous studies (references are cited in the manuscript) to achieve statistical significance of p<0.05 with 80% probability.
Data exclusions	No data were excluded
Replication	All the experimental findings were repeated with three independent experiments. All attempts at replication were successful.
Randomization	All animals were sex- and age-matched in the animal experiments as indicated in the figure legends. All the mice used in this study were randomly assigned to different treatments and respective control groups.
Blinding	The investigators were not blinded in this study because no bias could be made by the subject or the tester in the experiments performed.

Reporting for specific materials, systems and methods

We require information from authors about some types of materials, experimental systems and methods used in many studies. Here, indicate whether each material, system or method listed is relevant to your study. If you are not sure if a list item applies to your research, read the appropriate section before selecting a response.

Materials & experimental systems

n/a	Involved in the study	
☐	☒	Antibodies
☒	☐	Eukaryotic cell lines
☒	☐	Palaeontology and archaeology
☐	☒	Animals and other organisms
☒	☐	Human research participants
☒	☐	Clinical data
☒	☐	Dual use research of concern

Methods

n/a	Involved in the study	
☒	☐	ChIP-seq
☐	☒	Flow cytometry
☒	☐	MRI-based neuroimaging

Antibodies

Antibodies used	Anti-UCP1 (Abcam, #234430), anti-F4/80 (Abcam, #111101), Anti-throsine hydroxylase (THA) (Cell Signaling Technology, #58844), anti-α-smooth muscle actin antibody (Abcam, #124964), anti-monocyte/macrophage-2 (Abcam, #33451). Detailed information (supplier name, catalog number and clone name) of all antibodies used in this study are also described in the Methods and Materials section in Supplementary Information.
Validation	All antibodies used in this study are commercially available. The validation statements are available on the manufacturer's website.

Animals and other organisms

Policy information about studies involving animals; ARRIVE guidelines recommended for reporting animal research

Laboratory animals	Mouse, C57BL/6N, 8-week, male. Mouse, ApoE-/- mice on a C57BL6/J background, 8-week, male. Mouse, Adipo-MDM2-KO, 13-week, male.
Wild animals	The study did not involve wild animals.
Field-collected samples	The study did not involve samples collected from the field.
Ethics oversight	All animal experiments were approved by the Committee on the Use of Live Animals in Teaching and Research at the University of Hong Kong (Culatr No. is 4848-18).

Note that full information on the approval of the study protocol must also be provided in the manuscript.

Flow Cytometry

Plots

Confirm that:

- [x] The axis labels state the marker and fluorochrome used (e.g. CD4-FITC).
- [ ] The axis scales are clearly visible. Include numbers along axes only for bottom left plot of group (a 'group' is an analysis of identical markers).
- [ ] All plots are contour plots with outliers or pseudocolor plots.
- [x] A numerical value for number of cells or percentage (with statistics) is provided.

Methodology

Sample preparation	Stromal vascular fractions (SVFs) were isolated from iWAT of 6-week-old male C57BL/6 mice and were subjected to adipocyte differentiation by sequential treatment with 0.5 mM isobutylmethylxanthine, 2 µg/ml dexamethasone, and 10 ug/ml insulin for 48 hours, followed by treatment with 10 ug/ml insulin only for 6 days. Mature adipocytes at day 8 after differentiation were incubated with LCy5, PLCy5 with 2%, 5%, 10% of PTP at a Cy5 dose of 50 µmol/mL for 24 hours. In another group, cells were preincubated with PTP at a final concentration of 2 mmol/ml for 4 hours and followed by incubation with 5%PLCy5. Afterwards, the medium was removed and the cells were rinsed twice with PBS, followed by trypsinization and resuspension in PBS. Fluorescent intensity (Ex 650 nm, Em 670 nm) was determined by flow cytometry.
Instrument	Beckman Coulter, model CytoFLEX LX
Software	FlowJo software V10
Cell population abundance	The study did not involve cell sorting experiment
Gating strategy	The preliminary FSC/SSC gates of the starting cell populations were gated based on each cell population granularity and size. Dead cells and debris which have low FSC/SSC were excluded. Then using FSC-Height vs Area to enrich for single cells. Gating strategies were drawn according to untreated control vs nanoparticles treated cells to determine "negative" and "positive" cell populations.

- [ ] Tick this box to confirm that a figure exemplifying the gating strategy is provided in the Supplementary Information.
